# High dielectric permittivity sulfonyl-modified polysiloxanes as a dielectric for soft actuators†

**DOI:** 10.1039/d5tc01539a

**Published:** 2025-06-20

**Authors:** Cansu Zeytun Karaman, Thulasinath Raman Venkatesan, Johannes von Szczepanski, Frank A. Nüesch, Dorina M. Opris

**Affiliations:** a Functional Polymers, Empa, Swiss Federal Laboratories for Materials Science and Technology (EMPA) 8600 Duebendorf Switzerland Dorina.opris@empa.ch thulasinath.ramanvenkatesan@empa.ch; b Ecole Polytechnique Federale de Lausanne (EPFL) 1015 Lausanne Switzerland; c Eidgenössische Technische Hochschule Zürich (ETHZ) 8092 Zurich Switzerland

## Abstract

Dielectric elastomer actuators (DEAs) are soft transducers with great potential in soft robotics applications. Reducing the driving voltage and increasing the reliability of DEAs remain major challenges that need to be addressed. This can be achieved using thin dielectric elastomer films with high dielectric permittivity and low Young's modulus. Herein, high dielectric permittivity polysiloxanes were synthesized by functionalizing a polymethyvinylsiloxane with varying ratios of polar 3-mercaptosulfolane and mercaptobutyl groups, which allows for fine-tuning the dielectric permittivity of the resulting polymers. Thin films were cast using the doctor blade process and subsequently cross-linked *via* a UV-induced thiol–ene addition reaction to yield elastomers with a maximum dielectric permittivity of 15 at 1 Hz caused by the permanent dipole polarization. Differential scanning calorimetry shows a glass transition temperature (*T*_g_) below room temperature for all polymers. Dielectric impedance spectroscopy at different frequencies and temperatures revealed a secondary relaxation transition attributed to adsorbed water influencing the dielectric response of nearby polar groups. While such effects have been observed in other polymers, this is the first time they have been demonstrated in polar polysiloxanes. The polymer containing half of the repeat units modified by the sulfonyl group and half by butyl exhibited the most suitable dielectric and mechanical properties and, therefore, was further investigated as a dielectric in actuators. DEAs constructed from it can be operated over a wide voltage range and exhibit a lateral actuation strain of 7.2% at 14 V μm^−1^. Stack actuators constructed from this material exhibited an in-thickness actuation strain of 2.5% at 13.8 V μm^−1^ only, despite using a dielectric film with a thickness of 145 μm. Although our stack exhibits a lower dielectric breakdown field than stacks using acrylates or polydimethylsiloxane elastomers, it operates effectively at much lower electric fields. Since actuation scales with the inverse square of the dielectric thickness, reducing the thickness greatly enhances not only the actuation but also the breakdown field, supporting applications where low-voltage operation is critical.

## Abstract 2

High-permittivity elastomers were made by grafting sulfolane groups onto polysiloxane. Tuned polarity, thin films, and UV cross-linking yielded DEAs with 7.2% lateral strain at 14 V μm^−1^, enabling reliable low-voltage soft stack actuators.

## Graphical abstract

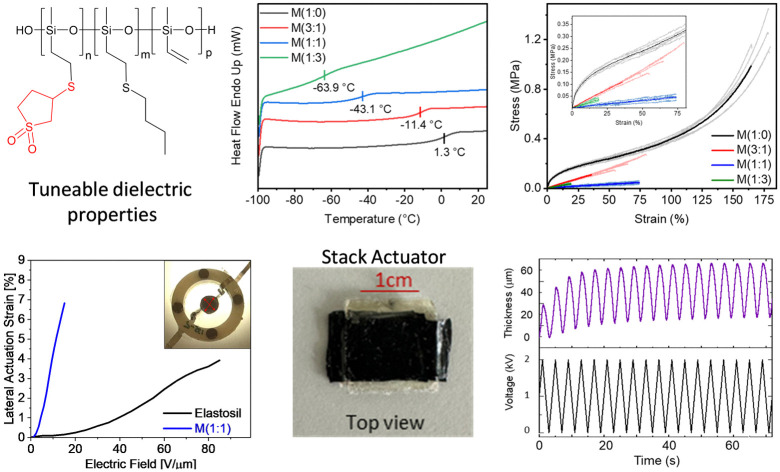

## 1. Introduction

**Fig. 1:**
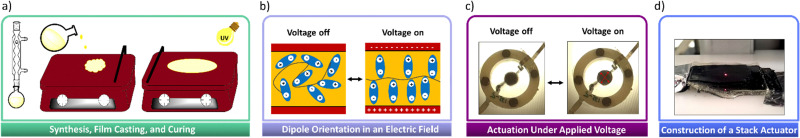
Steps involved in the process from synthesis to the construction of a dielectric elastomer actuator are illustrated: (a) synthesis of polysiloxane-based elastomers, casting into a thin film using doctor blade technique, and cross-linking; (b) behavior of the electric dipoles in an elastic network when the voltage is turned on and off; (c) actuation of single-layer dielectric elastomer actuator under a voltage; (d) photo of a stack actuator which thickness change under voltage is detected by a laser.

Research on high dielectric permittivity elastomers has increased significantly in recent years, as they are key components of many transducers.^1,2^ Dielectric elastomer actuators (DEAs) are stretchable capacitors that play a crucial role in various applications, including artificial muscles and skin, soft robotics, and flexible electronics.^3^ DEAs convert electrical energy into mechanical motion, which is attractive for electrically controlled actuation. However, the required voltages are typically in the kV range due to the low dielectric permittivity of most elastomers, which are nonpolar.^4^ DEAs operated at low voltages while giving large strains can be achieved using thin and soft dielectric films with high dielectric permittivity.^5–7^ Low-voltage DEAs require simpler electronics, which enhance energy efficiency and safe operation around humans.^8,9^ Additionally, the reduced material stress from operating at lower electric fields extends the life span of these actuators and may decrease the frequency of maintenance and replacement. These promising features make them applicable in various research areas, such as soft robotics, haptic feedback systems, and biomedical devices.^10,11^ DEAs can be constructed easily by coating a thin dielectric elastomer film with stretchable electrodes on both sides.^4^ Such capacitors are operated by charging and discharging them at a specific voltage and frequency.^12,13^ However, one of the greatest challenges in DEAs is to achieve high-dielectric permittivity (*ε*′) elastomers while not altering their softness and dielectric breakdown strength.^14,15^ So far, polyacrylates, polyurethanes, nitrile rubber, and polysiloxanes are the most explored elastomers in DEAs and have been incorporated into different actuators.^16–20^ Among polyacrylates, double-sided acrylic film adhesive from 3 M (VHB) is the most commonly used material. VHB shows an *ε*′ of 4.50–4.80 at 1 kHz and exhibits excellent electromechanical performance with actuation strains exceeding 380% in highly pre-strained films.^21,22^ However, VHB suffers from significant mechanical losses, leading to longer relaxation times and slower response rates.^23–25^ Other acrylate materials have also been reported, with some exhibiting outstanding actuation; however, relatively high voltages are still required.^22,26–28^ Polyurethanes have an *ε*′ of approximately 7 at 1 kHz.^29^ Despite their high dielectric permittivity, their actuation strain is relatively low due to the stiff nature of the elastomer.^30^ Polysiloxanes are characterized by outstanding flexibility and tunable modulus.^31,32^ They demonstrate moderate electromechanical actuation strain and low viscous losses, resulting in faster response times.^33,34^ Additionally, polysiloxanes can be operated over a wide temperature range and have low moisture absorption rates.^35,36^ However, their relative permittivity is low (*ε*′ = 2.5–3 at 1 kHz).^37^ Permittivity can be enhanced by grafting polar groups on the polysiloxane backbone, either through polymerizing monomers that contain polar groups or by attaching polar moieties to the backbone following polymerization.^38^ Various polar groups have been tested, such as trifluoropropyl (*ε*′ = 6.4), mercaptopropanamide (*ε*′ = 21), and nitrile (*ε*′ = 17.4).^38–40^ Recently, Sheima *et al.* synthesized a silicone elastomer bearing methylsulfone groups with a high relative permittivity of 23 and used this material to build DEAs that showed a lateral actuation strain of 14% at 24.2 V μm^−1^.^41^ This high performance is likely caused by the high polarizability of the sulfur atom coupled with two oxygen atoms, leading to dipole formation in an electric field.^42^ However, the glass transition temperature (*T*_g_) of this material is close to room temperature, impacting its elastic and dielectric properties. Here, we synthesized polysiloxanes starting from polymethylvinylsiloxane (PV), which was reacted with 3-mercaptosulfolane and mercaptobutane to give materials that exhibit a lower *T*_g_ than the one reported by Shaima *et al.* and, thus, a more stable actuation. The resulting polymers were cross-linked into thin films by exposing to UV light. The mechanical properties of the films were optimized, and their dielectric properties were investigated. Based on the experiments, the most promising material was selected and further investigated as a dielectric in single membrane actuators operated at different voltages and frequencies. Furthermore, a stack actuator was constructed, which could be operated at a low electric field. The steps involved, from polymer synthesis to actuator manufacturing and testing in a stack actuator, are illustrated in Fig. 1.

## 2. Results and discussion

### 2.1. Synthesis of materials

**Scheme 1:**
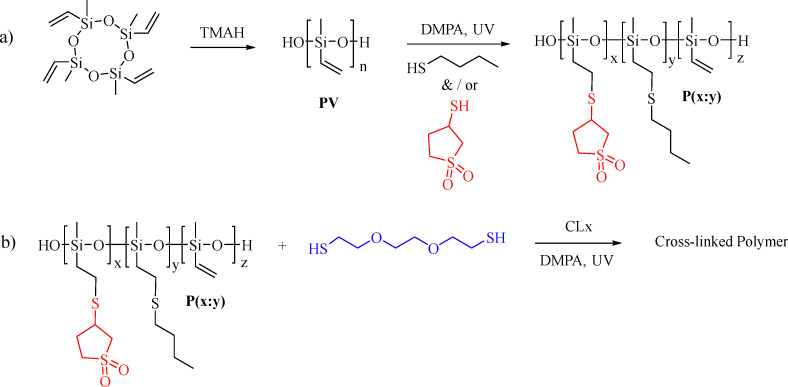
Synthesis of polymethylvinylsiloxane (PV) followed by grafting the thiols to PV (a) and cross-linking it by a UV-induced thiolene addition reaction with 2,2-dimethoxy-2-phenylacetophenone (DMPA) initiator (b).

**Fig. 2:**
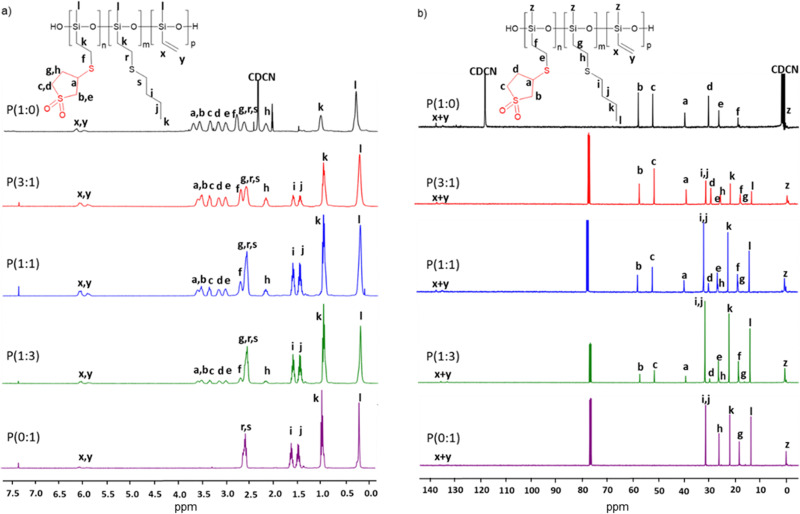
^1^H (a) and ^13^C (b) NMR spectra of the polymers P(*x* : *y*) in CDCl_3_ at room temperature.

Anionic ring-opening polymerization of 3,5,7-tetravinyl-1,3,5,7-tetramethylcyclotetrasiloxane was used for the synthesis of PV (for ^1^H and ^13^C NMR Fig. S1, ESI†) (*M*_w_ = 296 000 g mol^−1^, *M*_n_ = 193 900 g mol^−1^, PDI = 1.5, Fig. S2, ESI†). The vinyl groups on PV were used to graft polar groups on the backbone *via* UV-assisted thiol–ene click reaction (Scheme 1). Two different thiols, 3-mercaptosulfolane (Fig. S3 and S4, ESI†) and butanethiol, were grafted in various ratios, resulting in polymers denoted as P(*x* : *y*), where *x* : *y* represents the ratio of repeat units modified with 3-mercaptosulfolane (*x*) to butanethiol (*y*). Thus, we synthesized polymers P(1 : 0), P(3 : 1), P(1 : 1), P(1 : 3), and P(0 : 1). They are characterized by ^1^H and ^13^C NMR spectroscopy (Fig. 2) and GPC. The post-polymerization modification was conducted with less than stoichiometric amounts of thiols to the vinyl groups so that about 5 to 10 mol% of the vinyl groups were left unreacted on purpose. The significant decrease of the signals of the protons of the vinyl groups and the appearance of new signals caused by the reaction with the different thiols are clear evidence that the grafting was successful. Thermogravimetric analysis (TGA) provides insight into the thermal stability of the polymers. The starting polymer PV exhibits very good thermal stability, with only a slight mass loss at temperatures below 200 °C, likely due to the removal of cycle residues and a more pronounced degradation peak above 400 °C. By contrast, the grafted polymers exhibit lower thermal stability, with a minor weight loss of less than 0.5% below 100 °C, likely due to the evaporation of residual volatiles and the onset of decomposition beginning above 150 °C, possibly from the removal of cyclic contaminants. Above 250 °C, all polymers lose more than half of their weight, likely due to the decomposition of the grafted groups on the polysiloxane backbone (Fig. S5, ESI†).

**Fig. 3:**
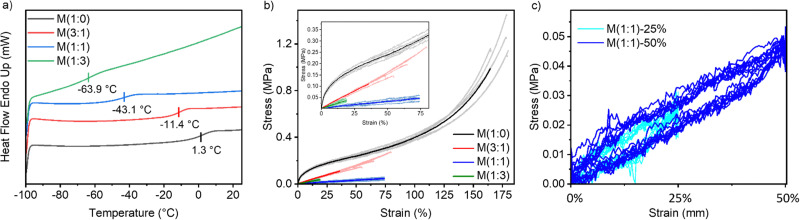
DSC measurements show the presence of a *T*_g_ for all materials synthesized (a). Stress–strain curves of the M(*x* : *y*) (b). Uniaxial cyclic test of M(1 : 1) at 25% and 50% strain (c).

The unreacted vinyl groups are subsequently used for cross-linking the polymers P(*x* : *y*) into thin films by 2′-(ethylenedioxy)diethanethiol cross-linker (Scheme 1). A homogeneous mixture of polymer, cross-linker, THF, 2,2-dimethoxy-2-phenylacetophenone (DMPA) UV active initiator was processed into thin films by doctor blading onto either a poly(vinyl alcohol) sacrificial layer or a Teflon substrate. After letting the THF evaporate, the films were exposed to UV light for 5 minutes. The obtained materials were denoted as M(1 : 0), M(3 : 1), M(1 : 1), and M(1 : 3) according to the starting polymer used for their synthesis. The optimum cross-linker concentration required to form films from all these materials was 0.307 mmol per 1 g polymers. The *T*_g_ of the obtained materials was determined by differential scanning calorimetry (DSC) (Fig. 3(a)). Increasing the concentration of 3-mercaptosulfolane in M(*x* : *y*) led to an increase in *T*_g_, bringing it closer to room temperature while increasing the concentration of butanethiol resulted in a decrease in *T*_g_. This phenomenon is likely due to the bulky nature of the 3-mercapto sulfolane group, which increases steric hindrance. In contrast, the long alkyl chain of butanethiol interrupts the intermolecular interactions, increasing flexibility and reducing *T*_g_. TGA investigation showed that the materials are stable up to 150 °C (Fig. S5b, ESI†).

### 2.2. Mechanical characterization

The stress–strain curves of the cross-linked materials were investigated by tensile test (Fig. 3(b)), and their Young's modulus is reported at 10% strain (Table 1). From all materials, M(1 : 0) had the highest strain at break, with an average of 168%, compared to 64% for M(3 : 1), 73.5% for M(1 : 1), and 19% for M(1 : 3). M(3 : 1) and M(1 : 1) showed similar strain at break with different Young's modulus of 333 kPa and 75 kPa, respectively. M(3 : 1) is stiffer than M(1 : 1) and can be due to its higher content of the bulky polar group, which causes an increase in the *T*_g_, which is closer to room temperature. Both M(1 : 1) and M(3 : 1) exhibit reversible deformation with minimal hysteresis between cycles in uniaxial cyclic tests at two different strain levels of 25 and 50% (Fig. 3(c) and Fig. S6a, ESI†). The most polar material M(1 : 0) shows inelastic deformation, which can be clearly seen in the uniaxial cyclic test (Fig. S6b, ESI†).

**Table 1:** Key parameters of the materials synthesized: *T*_g_ – glass transition temperature obtained from DSC, *s* – maximum strain at break, *Y*_10%_ – Young's modulus at 10% strain, *E*′ – storage modulus, tan *δ* – dissipation factor, and *ε*′ – dielectric permittivity

Material	Cross-linker [mmol]	Thickness [μm]	*T*_g_ [°C]	*s* [%]	*Y*_10%_ [kPa]	*E*′ @ 0.05 Hz [kPa]	tan *δ* @ 0.05 Hz	*ε*′ @ 1 kHz
M(1 : 0)	0.307	105	1.3	168 ± 22	991 ± 22	206	0.95	6.8
M(3 : 1)	0.307	102	−11.4	64 ± 11	333 ± 7	146	0.13	6.6
M(1 : 1)	0.307	121	−43.1	73.5 ± 0.5	75 ± 18	250	0.008	9.2
M(1 : 3)	0.307	135	−63.9	19 ± 0.1	187 ± 45	290	0.002	7.8

**Fig. 4:**
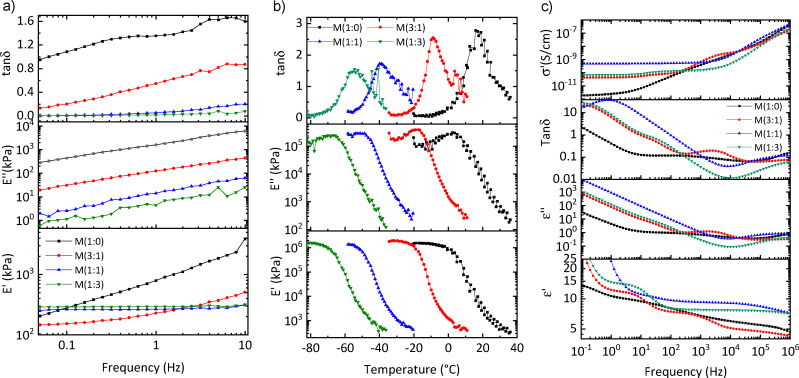
DMA of the materials at 2% strain at room temperature (a) and different temperatures (b). The conductivity (*σ*′), loss tangent (tan (*δ*)), and dielectric permittivity (*ε*′) at room temperature as a function of frequency ranging from 10^−1^ and 10^6^ Hz (c).

Dynamic mechanical analysis (DMA) was conducted to determine the storage modulus (*E*′), loss modulus (*E*′′), and tan *δ* of the materials across a frequency range from 0.05 Hz to 10 Hz at 2% strain, both at room temperature and varying temperatures (Fig. 4(a) and (b)). The storage modulus of M(1 : 1) and M(1 : 3) was almost constant at different frequencies, whereas M(1 : 0) and M(3 : 1) exhibited a significant increase in *E*′ with increasing frequency. Tan *δ* provides information about the mechanical behavior of the materials. M(1 : 0) and M(3 : 1) demonstrate significant mechanical losses at elevated frequencies. In contrast, M(1 : 1) and M(1 : 3) exhibit tan *δ* values below 0.2 at 10 Hz, indicating minimal mechanical losses. The mechanical losses of the more polar material M(1 : 0) are rather high, even at low frequencies, which makes them unattractive for actuator applications. The DMA and tensile and uniaxial cyclic test results allow us to identify a material M(1 : 1) with attractive mechanical properties for actuators. It is soft, with a low elastic modulus, and exhibits reversible deformation with minimal mechanical losses.

Temperature-dependent DMA provides information about the transition temperatures occurring in a material (Fig. 4(b)). *T*_g_ values can be clearly observed at the maximum in tan *δ* peak, where the curve undergoes the most significant change with temperature. For the materials examined, *T*_g_ values are as follows: 2.5 °C for M(1 : 0), −17.2 °C for M(3 : 1), −48.6 °C for M(1 : 1), and −70.32 °C for M(1 : 3), respectively. These values are quite similar to those found in DSC measurements.

### 2.3. Dielectric characterization

Dielectric impedance spectroscopy offers insights into different polarization mechanisms at varying frequencies. The materials are subjected to a small alternating voltage (1 V) during which the dielectric permittivity (*ε*′), the dielectric loss (*ε*′′), and the tan *δ* of the materials are measured over a frequency range from 0.1 Hz to 1 MHz. (Fig. 4(c)). For all materials, *ε*′ values at 1 kHz were summarized in Table 1. The dielectric permittivity of all materials exhibited a very strong dependency on frequency. Material M(1 : 0) has the highest content of polar sulfonyl group. Despite the presence of polar groups in the material and their potential to enhance dielectric permittivity through dipolar polarization, the observed dielectric permittivity remained low at high frequencies and only gradually increased at lower frequencies. The reason behind the low permittivity is the rather high *T*_g_ of M(1 : 0), which approaches room temperature. Therefore, the dipoles are frozen and cannot contribute to the permittivity, even at low frequency. Material M(3 : 1), exhibited a low dielectric permittivity at frequencies above 10 kHz, which indicates that the dipoles cannot follow the electric field. However, at lower frequencies, three processes can be distinguished. As will be discussed later, the low-frequency process arises from electrode polarization, whereas the other processes are caused by dipole polarization. The same polarization processes are observed for material M(1 : 1), however, their relaxation frequency is shifted to higher frequencies. One has a relaxation frequency above 1 MHz, while the second has a relaxation at about 10 Hz. Only the dipoles of M(1 : 3), which *T*_g_ = −73 °C, were sufficiently mobile at all frequencies, and, therefore, its dielectric permittivity at room temperature and frequencies reached values above 10 at all frequencies. At low frequency below 0.5 Hz, electrode polarization is observed.

**Fig. 5:**
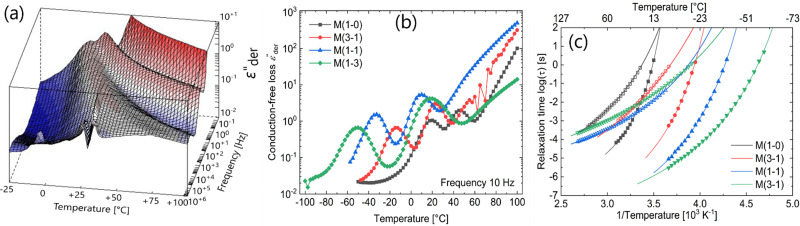
Dielectric impedance measurements on the various samples: 3D plot of conduction-free dielectric loss (
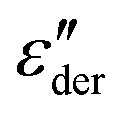
) of a M(1-0) sample (a), 
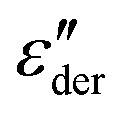
 as a function of temperature at 10 Hz (b), and Arrhenius relaxation plot with open symbols for VFT fit 1 and closed symbols for VFT fit 2 (c).

Temperature-dependent impedance measurements can complement the results from DSC and DMA as they also reveal the various phase transitions occurring in a dielectric sample, as seen from the changes in the real and imaginary parts of permittivity. Its higher sensitivity and scope for additional analysis can reveal additional processes not observed in other thermal and mechanical measurements.^43^ To investigate the various relaxations in the modified polysiloxanes in detail, dielectric measurements were conducted as a function of temperatures and frequencies. (Fig. S7–S10, ESI†). From Fig. 4(c), at lower frequencies (around 10 Hz), we observe an additional step in permittivity. However, we do not observe a corresponding loss peak due to the onset of electrode polarization. To visualize the low-frequency processes more clearly, the contribution from d.c. conductivity was removed using a derivative technique introduced by Wübbenhorst and van Turnhout.^44,45^Fig. 5(a) shows the 3-dimensional plot of the d.c. conduction-free dielectric loss (
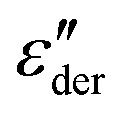
) of M(1-0) film as a function of temperature and frequency. 
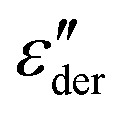
 can be obtained from the frequency derivative of *ε*′ as stated in eqn (1), where *ω* denotes angular frequency.1
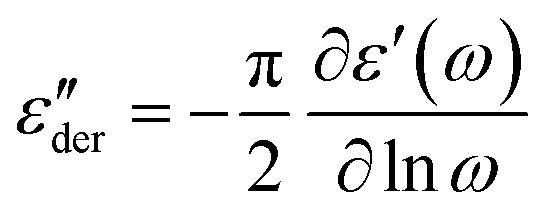


From Fig. 5(a), two relaxation processes with a characteristic shift to higher temperatures with an increase in frequency can be identified. In addition, a steep increase in the losses is observed at higher temperatures and lower frequencies, indicative of electrode polarization. The absence of the latter process in the 3D representation of electric loss modulus (M′′) (Fig. S11, ESI†) confirms the presence of electrode polarization.^43,46^ The 
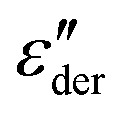
 curves of the other materials at 10 Hz plotted in Fig. 5(b) as a function of temperature reveal the existence of dual relaxations in all samples.

The two relaxation processes were subjected to a Havriliak–Negami (HN) fit commonly used to analyze non-Debye relaxations in polymer materials. The resultant relaxation times for all the samples are plotted in an Arrhenius plot in Fig. 5(c). Both relaxation processes follow a non-linear Vogel–Fulcher–Tammann (VFT) behavior with temperature. Hence, they represent a glass-transition relaxation. The *T*_g_s can be calculated as the temperature at which the relaxation time is 100 s (log *τ* = 2 s).^47^ The calculated values of all the samples from Fig. 5(c) are summarized in Table 2.

**Table 2:** Fitted parameters and calculated *T*_g_s from impedance fitting

Sample	*T*_g_ 1 [°C]	Activation energy [kJ mol^−1^]	*α*	*β*	*T*_g_ 2 [°C]	Activation energy [kJ mol^−1^]	*α*	*β*	Δ*T*_g_ [°C]
M(1-0)	+06.7	4.89	0.46	0.51	+07.7	9.84	0.88	0.90	1
M(3-1)	−25.6	7.35	0.45	0.48	−18.7	15.63	0.91	1.00	7
M(1-1)-200	−45.4	8.34	0.48	0.51	−30.4	8.32	0.94	0.97	15
M(1-3)	−63.9	7.80	0.52	0.38	−38.4	11.87	0.93	1.00	26

The two *T*_g_s of the most polar material M(1-0) are very close to each other, and from the Arrhenius plot, we observe that these transitions superimpose at lower frequencies. A similar phenomenon of dual glass-transitions was previously observed in thiol-modified polysiloxanes (both homo- and co-polymers) prepared by our group.^48^ In the case of materials M(3-1) to M(1-3), we observed that an increase in the butyl group content widens the difference between the two *T*_g_s. The presence of a 2^nd^*T*_g_ relaxation in M(1-0) homopolymer made us consider that the phase segregation process may be responsible for this. However, further experiments did not confirm this. Similar to M(1-0), the material containing only butyl thioether M(0-1), also shows a dual glass transition behavior (Fig. S12, ESI†).

The similar activation energies of the 2^nd^ relaxation in both homo- and co-polymer samples suggest the common origin of this phenomenon (Table 2). Looking at the HN fitting parameters *α* and *β*, they are close to 1. This points to a Debye-like relaxation observed in materials with a single relaxation time, such as small molecules.^49^ Both cycles and water can be present in the elastomers, which are possible sources for this 2^nd^ transition. Cycles are always present in any polysiloxane materials as their formation cannot be prevented during synthesis, and if their molar mass is high, they cannot be easily removed by vacuum distillation. To investigate if the cycles are responsible for this, 1,3,5,7-tetravinyl-1,3,5,7-tetramethylcyclotetrasiloxane monomer was functionalized with the sulfonyl and butyl groups, respectively, and the resulting functionalized monomers were measured by DRS. Since the functionalized monomers are pure, only one *T*_g_-relaxation is expected. However, their 
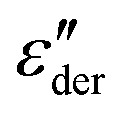
 plots, similar to that of M(*x* : *y*), reveal the presence of two non-linear relaxations, as shown in Fig. S13 (ESI†). Hence, we hypothesize the 2^nd^*T*_g_ is due to water molecules present in the elastomer. These water molecules can interact with the polar groups and modify their dielectric behavior, leading to a delayed relaxation.

Panagopoulou *et al.* reported a similar dual *T*_g_ phenomenon with protein–water mixtures.^50^ DRS and thermally stimulated depolarization currents (TSDCs) measurements allowed detect an additional glass transition relaxation even at low water contents. They suggested that microphase segregation occurs due to the different degrees of plasticized protein chains. The primary *T*_g_ was associated with the more plasticized regions, which undergo a combined relaxation of the dipoles and the water molecules interacting with it. They suggested that the water molecules are either sorbed to the dipoles polar or form clusters that interact with the protein surface. Above a certain critical water content, a separate phase of water clusters forms in certain regions, which percolates and interconnects protein chains. These regions are less plasticized, which leads to a delayed dielectric response, ultimately resulting in an additional *T*_g_. Similarly, the modified elastomers can have regions with different degrees of plasticization, leading to two *T*_g_s. The observation of higher losses associated with the 2^nd^ glass transition in comparison to the primary *T*_g_ in both cases also supports this hypothesis (Fig. 5(b)). Considering the above hypothesis, from Fig. 5(b), (c) and Table 2, we can infer the difference in the degree of plasticization in the samples with different polarity. While the difference between the two *T*_g_s is minimum for the most polar M(1-0) material, it is maximum for the least polar M(1-3) material. This can be explained by the stronger interaction of water molecules in the more polar material M(1 : 0) with high sulfonyl group content, resulting in a stronger water-reinforced sample than materials with a higher amount of weakly polar butanethiol groups. To remove the absorbed water, the samples were heated to 140 °C for several hours in a vacuum oven and subjected to dielectric measurements. Fig. S15 (ESI†) compares the 
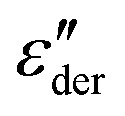
 curves of a M(1-0) homopolymer sample before and after annealing. While we observe a decrease in the overall water content apparent from the lower losses of the annealed sample and a shift in the *T*_g_ peaks to a higher temperature, we can also infer that the water molecules are bound to the polar groups since we still see a 2^nd^*T*_g_ peak. A similar trend can be seen in the other samples, as shown in Fig. S16–S18 (ESI†). Hence, it is clear that the water clusters are tightly bound with the polar groups belonging to the same or different chains and cannot be completely removed from M(*x* : *y*) even after annealing at elevated temperatures.

The less polar M(1-1) and M(1-3) materials do not show significant changes in the primary *T*_g_ loss peak height and temperature after annealing (Fig. S17 and S18, ESI†). In the case of these elastomers with lower sulfonyl content, as mentioned earlier, they will be reinforced less compared to their high polar counterparts. This leads to a scenario where the absorbed water cluster primarily interacts with the sulfonyl groups spread across different polymer chains (interchain cluster formation), leading to a strong secondary *T*_g_ relaxation, while the primary *T*_g_ remains unaltered.^51^ The observed higher losses of the secondary *T*_g_ relaxation of low polar M(1-1) and M(3-1) samples in comparison to more polar M(1-0) and M(3-1) samples in Fig. 5(b) supports this hypothesis.

### 2.4. Electromechanical properties

**Fig. 6:**
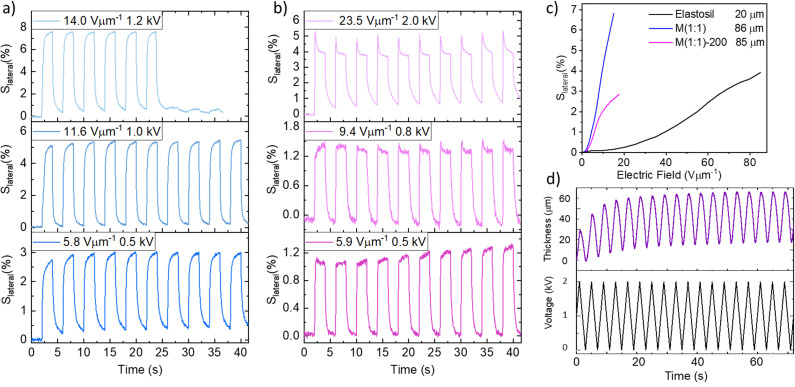
Lateral actuation strain of M(1 : 1) for 10 actuation cycles at 0.25 Hz at 5.8 V μm^−1^, 11.6 V μm^−1^, and 14 V μm^−1^ (a). Lateral actuation strain of M(1 : 1)-200 for 10 actuation cycles at 0.25 Hz at 5.9 V μm^−1^, 9.4 V μm^−1^, and 23.5 V μm^−1^ (b). Lateral actuation strain at different electric fields of M(1 : 1), M(1 : 1)-200, and Elastosil (c). The change in thickness of a stack actuator constructed from M(1 : 1)-200 operated at 2 kV and 250 mHz for 18 cycles (d).

A suitable dielectric elastomer for an actuator should exhibit good elastic properties with minimal mechanical losses, Young's modulus below 1 MPa, and a high dielectric permittivity. Among all materials tested, M(1 : 1) exhibits the best mechanical properties alongside the highest *ε*′ at room temperature and, therefore, was further investigated as dielectric in actuators. An actuator constructed from an 86 μm thick film gave 2.6% lateral actuation strain at 5.8 V μm^−1^ (Fig. 6(a)), and its lateral actuation increased to 5.3% at 11.6 V μm^−1^. The actuator showed stable and reversible actuation with no hysteresis between cycles. This behavior is consistent with the results from the uniaxial cyclic test, which show that the material exhibits minimal hysteresis, indicating low energy and mechanical losses. The actuation reached a maximum of 7.2% at 14 V μm^−1^. At 14 V μm^−1^, the actuation was stable for only six cycles, after which the actuator suffered a breakdown. The low dielectric breakdown field is also confirmed by the Weibull probability plot (Fig. S19a, ESI†), which gives the statistical distribution of dielectric strength. It offers valuable insights into the material's reliability as a dielectric. For each material, 10 individual samples were tested to ensure statistical relevance. The samples were prepared under the same conditions before conducting measurement and sample thickness, breakdown voltage, and breakdown field for the materials summarized (Tables S2 and S3, ESI†). The plot shows a slope parameter (*β*_b_) of 6.7 and a scale parameter of 11.7 V μm^−1^ corresponding to the breakdown field, at which 63.2% of the samples fail. It represents the maximum operating electric field for the actuator.

A material with a higher elastic modulus withstands higher voltages, therefore, a stiffer material was synthesized starting from P(1 : 1), but the amount of cross-linker used was increased four times. The resulting material was M(1 : 1)-200 and its mechanical and dielectric characterizations are included in (Fig. S20, S21 and Table S2, ESI†). It is stiffer than M(1 : 1) with an elastic modulus of *Y*_10%_ = 430 kPa, but the Weibull probability plot shows that it is also more reliable than M(1 : 1), having a slope parameter of 3.9 and a scale parameter of 30 V μm^−1^ (Fig. S19b and S20, ESI†). As expected, at the same electric field, M(1 : 1)-200 shows lower actuation than M(1 : 1). This is due to its higher cross-linking density, which makes it stiffer, thus requiring higher voltages for actuation. The M(1 : 1)-200 based actuator with a thickness of 85 μm responded to a rather low electric field of 5.9 V μm^−1^ with 1.2% lateral strain (Fig. 6(b)) and, at 9.4 V μm^−1^, exhibited an actuation strain of 1.7%. This actuator withstood an electric field of 23.5 V μm^−1^ (2 kV) and displayed 4.7% actuation, the highest measured for these materials. Additionally, the actuator exhibited stable and reversible actuation over 100 cycles at 1.5 kV (Fig. S22a, ESI†).

Both M(1 : 1), and M(1 : 1)-200 materials are compared with Elastosil film with 20 μm thickness in terms of lateral actuation strain at different electric fields (Fig. 6(c)). Both materials showed much higher actuation strains at lower electric fields compared to Elastosil, even though Elastosil is thinner. However, Elastosil can withstand much higher voltages of 85 V μm^−1^. Therefore, our materials are promising for low electric field responsive applications.

### 2.5. Stack actuator

Because of the higher dielectric breakdown field of M(1 : 1)-200, this material was selected for constructing the stack. A previously reported material was used as an electrode material.^52^ It was melt-pressed in thin film. The stack comprises five active M(1 : 1)-200 layers with a thickness of 145 μm and six electrode film layers with a thickness of 205 μm (Fig. S23, ESI†). The stack actuator had a thickness of 1955 μm. The actuator was evaluated at 2 kV at a frequency of 0.25 Hz (Fig. 6(d)). When a voltage is applied, the electrostatic pressure from the electrodes compresses the elastomer films in the cross-plane direction, causing a reduction in the stack's thickness. When the voltage is turned off, the actuator returns to its original shape. The actuator displayed an average thickness change of 49 μm, equivalent to an actuation of 2.5% at an electric field of 13.8 V μm^−1^. The stack was operated for over 1000 cycles, and after about 180 cycles, it suffered several breakdowns (Fig. S22b, ESI†). Nevertheless, it was able to self-heal multiple times and continued to function. The electrostatic pressure (*p*) and the force (*F*) generated by the stack actuators with a 1 cm^2^ active area are also summarized (Table 3). The force produced by the devices can be calculated using the formula *F* = *p*·*n*·*A*, where *F* is the force, *p* is the Maxwell pressure, *p* = *ε*_0_*ε*′*E*^2^, with *ε*_0_ representing the vacuum permittivity, *ε*′ the relative permittivity, and *E* the electric field. The calculated force generated by a 5-layer actuator was 7.4 N at an electric field of 13.8 V μm^−1^. Although acrylates and polydimethylsiloxane elastomers have been studied in stack actuators, most of these devices require a relatively high electric field in order to actuate. While high-permittivity polysiloxane elastomers have shown promise in single-layer actuators, their integration into stacked configurations is still largely unexplored. Our single-layer actuators exhibited a lateral actuation strain of 7.2% at 14 V μm^−1^, while stack actuators reached 2.5% strain at 13.8 V μm^−1^. In contrast, chloromethyl-functionalized polysiloxanes reported in the literature exhibit 4.6% lateral strain at 11.2 V μm^−1^ and 1.0% actuation in a stack at 12.4 V μm^−1^. Since actuation scales with the inverse square of the dielectric thickness, reducing the thickness significantly enhances both actuation and the breakdown field, supporting applications where low-voltage operation is critical. This will be an important direction for future work in our group. Opris’ group prepared a series of polymers with different polarities and high dielectric permittivity.^38^ Several of these were successfully cross-linked into elastomers, and their actuation performance was investigated.^1^ Expanding this library further, with a wider variety of polar groups, holds great potential for uncovering how actuation properties can be optimized. While the materials we report here may not yet surpass those published previously by our group,^5^ these results provide valuable insights and a strong foundation for designing improved materials. Through careful investigation and a deeper understanding of how structure influences properties, we are confident that more advanced materials can be developed.

**Table 3:** Actuation properties of the stack

	*d* [μm]	Δ*d* [μm]	*V* [kV]	*E* [V μm^−1^]	*S*_z_ [%]	*P* [kPa]	*A* [cm^2^]	No. layers	*F* [N]
Stack	145	49	2.0	13.8	2.5	14.8	1.0	5	7.4

## 3. Conclusions

In this study, we synthesise of polysiloxanes modified with different content of sulfonyl groups. For their synthesis, we used polymethylvinylsiloxane and conducted a post-polymerization modification using different amounts of 3-mercaptosulfolane and butanthiol. The polymers’ dielectric properties and *T*_g_ can be easily tuned by varying the polar sulfonyl to butyl group content. DSC measurements show that all polymers exhibit a *T*_g_ below room temperature, which is a must to achieve elastomers after cross-linking. The unreacted vinyl groups were subsequently used for cross-linking into thin films by a thiol–ene click reaction, which led to elastic materials. Dielectric relaxation spectroscopy investigations revealed the presence of a second glass transition relaxation above the primary relaxation. The presence of water molecules that interact with the polar groups of the polysiloxanes is hypothesized as the reason for this dual *T*_g_. The observed 2^nd^*T*_g_ depends on the sample's polarity and hence varies with the amount of sulfonyl groups. Annealing reduces the strength of the 2^nd^*T*_g_, however, it cannot be eliminated even when the samples are dried at high temperatures and vacuum. The detailed dielectric investigation shows the impact of moisture on the dielectric of polar polysiloxanes. The cross-linked M(1 : 1) elastomer exhibited the highest dielectric permittivity of 9.3 at room temperature and the best elastic properties with minimal mechanical losses. Hence, actuators were constructed from this material. It showed an actuation strain of 7.2% at 14 V μm^−1^. To further improve the actuation, another material was synthesized for which the cross-linker amount was quadrupled, showing an actuation strain of 4.7% at 23.5 V μm^−1^. Compared to Elastosil, both materials exhibited much higher actuation strains at low electric fields. Finally, stack actuators constructed from M(1 : 1)-200, consisting of five dielectric layers and six electrode layers, showed a 2.5% thickness actuation at 13.8 V μm^−1^. Due to the facile synthesis of the starting materials, including the thiol used for functionalization, the synthesis of this material can be easily scaled up. Therefore, future work will focus on increasing the processability of these materials so that films less than 20 μm thick are achievable, and their dielectric breakdown field will be increased, allowing access to powerful stack actuators operated at lower voltages.

## 4. Experimental

### 4.1. Materials

Unless otherwise mentioned, all chemicals were used without further purification. 3-Sulfolene, toluene, methanol, dichloromethane, tetrahydrofuran, sodium bicarbonate (NaHCO_3_) and 2,2-dimethoxy-2-phenylacetophenone (DMPA) were purchased from VWR. Thioacetic acid, tetramethylammonium hydroxide (TMAH), butanethiol, 2,2′-(ethylenedioxy)diethanethiol, and benzene were purchased from Sigma-Aldrich. Additionally, trimethylsilyl chloride (TMSCl) and 1,3,5,7-tetravinyl-1,3,5,7-tetramethylcyclotetrasiloxane were supplied by ABCR. PV was prepared according to the literature.^38^ The Elastosil® Film 2030, used in this work, is produced by Wacker. It exhibits excellent mechanical and physical properties. According to the manufacturer, the films are manufactured under Class 8 cleanroom conditions to ensure high purity and consistent quality.

### 4.2. Characterization

^1^H and ^13^C NMR spectra were measured on a Bruker AV-III 400 spectrometer (Bruker BioSpin AG, Switzerland), placing samples in a 5 mm CryoProbe™ Prodigy probe at 400.2, and 100.6 MHz for ^1^H and ^13^C NMR, respectively. All NMR experiments were taken at 298 K using a Bruker standard pulse program and parameter sets. ^1^H and ^13^C NMR chemical shifts (*δ*) were calibrated to residual solvent peaks.

Tensile and cyclic uniaxial stress tests were conducted using a Zwick Z010 testing machine, operating at a crosshead speed of 50 mm per minute. Test specimens for the tensile tests were prepared with a gauge width of 2 mm and a gauge length of 18 mm using a bone-shaped die-cutting method. Strain measurements were obtained using a traversing sensor. The resulting curves were averaged from five samples of each material using Origin software, and the tensile modulus was determined from the slope of the stress–strain curves, obtained by a linear fit to the data points from 0 to 10% strain. Five samples of each material were tested for tensile strength, while one sample of each material was used for cyclic tensile testing.

Dielectric permittivity measurements were performed with a Novocontrol Alpha dielectric analyzer, while sample temperature was regulated by a Novocontrol Quatro cryosystem in a dry nitrogen environment. The prepared uniform films were sandwiched between two stainless steel electrodes. The dielectric permittivity of the films was measured while ranging in frequency from 10^−1^ to 10^6^ Hz. For each material, impedance spectroscopy was performed on three samples at room temperature and one sample at varying temperatures.

DMA measurements were performed by a RSA 3 DMA from TA Instruments. Uniform films were cut with a width of 10 mm and a length 10 mm stripes and were measured under 2.5 g dynamic load at 2% strain, while frequency was scanned between 0.05 and 10 Hz at 298 K. Three samples of each material were used for room-temperature testing, and one sample of each material was tested for temperature-dependent measurements.

DSC investigations were taken place on a PerkinElmer Pyris Diamond DSC apparatus. Approximately 10 mg of the sample was accurately weighed and placed in aluminum crucibles sealed with perforated lids. Each measurement involved two heating cycles and one cooling cycle, with a heating and cooling rate set at 10 °C min^−1^, spanning temperatures from −60 to 100 °C, all performed under a nitrogen atmosphere. The second cooling cycle was utilized to determine the *T*_g_.

TGA was investigated using a PerkinElmer TGA7 with a heating rate of 10 °C min^−1^ under a nitrogen gas flow from 25 to 700 °C.

GPC measurements in tetrahydrofuran were performed using an Agilent 1260 infinity on two tandem-connected mixed-bed columns (1 × PLgel 5 μm MIXED-C Guard and 2 × PLgel 5 μm MIXED-C Analytical) and a 390-MDS refractive index detector. The flow rate was 1 mL min^−1^, and the temperature was 35–40 °C. PS standards were used for calibration.

### 4.3. General procedure for the functionalization of PV with polar thiols

PV (9 g) was dissolved in distilled THF (100 mL). To this solution, butanethiol and/or 3-mercaptosulfolane followed by DMPA (0.22 g, 0.9 mmol, 0.01 eq.) were added under an argon atmosphere. After three freeze–thaw–pump cycles were applied, the mixture was exposed to UV light for 30 min. After removing the solvent under reduced pressure, the mixture was precipitated with methanol. The precipitate was dissolved in a minimum amount of THF and precipitated again with methanol. This procedure was repeated several times until all impurities were removed. After vacuum drying, the pure polymers were obtained.

### 4.4. Cross-linker solution preparation

2,2′-(ethylenedioxy)diethanethiol (0.45 g, 400 μL) was weighed in a vial and dissolved in THF (2000 μL) to obtain a solution of 1 : 5 (v : v) of cross-linker in THF. Each elastomer was prepared from a fresh cross-linker solution stored in a brown bottle.

### 4.5. Thin film formation

A homogenous mixture of polymer (1 g), DMPA initiator (4.5 mg) in a minimum volume of THF (1 mL), cross-linker solution in THF (1 : 5) 50 μL solution for M(1 : 0), M(3 : 1), M(1 : 1), M(1 : 3), and 200 μL for M(1 : 1)-200 was prepared using a speed mixer for 5 minutes at 3000 rpm. Subsequently, the mixture was blade-cast onto either a Teflon substrate or a poly(vinyl alcohol) sacrificial layer, previously cast on a glass substrate, and left overnight in a laminar flow. The films were exposed to UV irradiation for 5 minutes to yield cross-linked elastic films, which were stored in a vacuum oven at 60 °C for at least 1 day before further characterization.

### 4.6. Actuator construction

The films were biaxially pre-strained by 5% and fixed between two circular rigid plastic frames to sustain this pre-strain. Circular electrodes of carbon black powder with a diameter of about 8 mm, were drawn on both sides of the film and connected to a high-voltage source *via* aluminum strips. Actuation strain was optically measured by observing the extension of the electrode area's diameter using a digital camera. A LabView program, incorporating an edge detection tool, was employed to determine the boundary between the black electrode area and the transparent silicone film.

### 4.7. Stack actuator construction

A stack actuator was constructed by layering an interdigitated structure composed of alternating 5 dielectric films (145 μm thick and a size of 1 × 1.5 cm) and 6 electrode layers (205 μm thick and a size of 1 × 1.5 cm). The elastic electrodes were synthesized through anionic ring-opening polymerization with tetramethylammonium hydroxide as an initiator of a cyclic silicone monomer bearing cyanopropyl functional groups, combined with a polar bicyclic cross-linker, enabling one-step polymerization and cross-linking. The actuator was assembled by alternately stacking the dielectric films and electrode layers, ensuring each active area measured 1 cm^2^. The assembled device was then placed on a 100 °C hot plate for 1 hour, followed by overnight curing in a vacuum oven at 60 °C. The final device had a total thickness of 1.95 mm. Its thickness variation during actuation was precisely measured using a laser.

## Author contributions

C. Z. K. synthesized all polymers and materials and characterized polymers. T. R. V. conducted the dielectric relaxation spectroscopy investigations. J. S. conducted the DMA measurements, C. Z. K. conducted the tensile test measurements. D. M. O. conducted actuator measurements. C. Z. K., T. R. V., and D. M. O. wrote the original draft. D. M. O. initiated the activity, designed the materials, received funding acquisition, and coordinated and supervised this research. All authors contributed with discussions, reviewing, and editing and have approved the final version of the manuscript.

## Conflicts of interest

There are no conflicts to declare.

## Supplementary Material

TC-013-D5TC01539A-s001

## Data Availability

The data supporting the findings of this study are available within the article and its ESI.† The raw data generated and analyzed during the current study are available from the corresponding author upon reasonable request. All raw data were uploaded in Zenodo: 10.5281/zenodo.15223936 and will be made available on request.
