# Supplementary material for: High dielectric permittivity sulfonyl-modified polysiloxanes as a dielectric for soft actuators
Source: J Mater Chem C Mater. 2025 Jun 20;13(31):15886–96. doi: 10.1039/d5tc01539a (PMC12218110; doi:10.1039/d5tc01539a)
Supplement: TC-013-D5TC01539A-s001 [file TC-013-D5TC01539A-s001.pdf]

Supplementary Information (SI) for Journal of Materials Chemistry C.
This journal is © The Royal Society of Chemistry 2025

High Dielectric Permittivity Sulfonyl-Modified Polysiloxanes as a Dielectric for Soft Actuators

Cansu Zeytun Karaman[a,b], Thulasinath Raman Venkatesan[a], Johannes von Szczepanski[a,c], Frank A. Nüesch[a,b], and Dorina M. Opris[a,c]**

[a]*Functional Polymers,Empa, Swiss Federal Laboratories for Materials Science and Technology (EMPA), 8600 Duebendorf, Switzerland.*

[b]*Ecole Polytechnique Federale de Lausanne(EPFL), 1015 Lausanne, Switzerland*

[c]*Eidgenössische Technische Hochschule Zürich (ETHZ)), 8092 Zurich, Switzerland*

E-mail: thulasinath.ramanvenkatesan@empa.ch; dorina.opris@empa.ch

Table of Content

GPC...2

Synthesis...3

NMR Spectra...4

TGA...5

FTIR...5

Uniaxial Cyclic Test of M(1:0) and M(3:1)...6

Tensile Test and DMA of M(1:1)-200...7

Temperature Dependent Dielectric Permittivity Measurements...7

Dielectric Breakdown...9

Actuation test...9

NMR

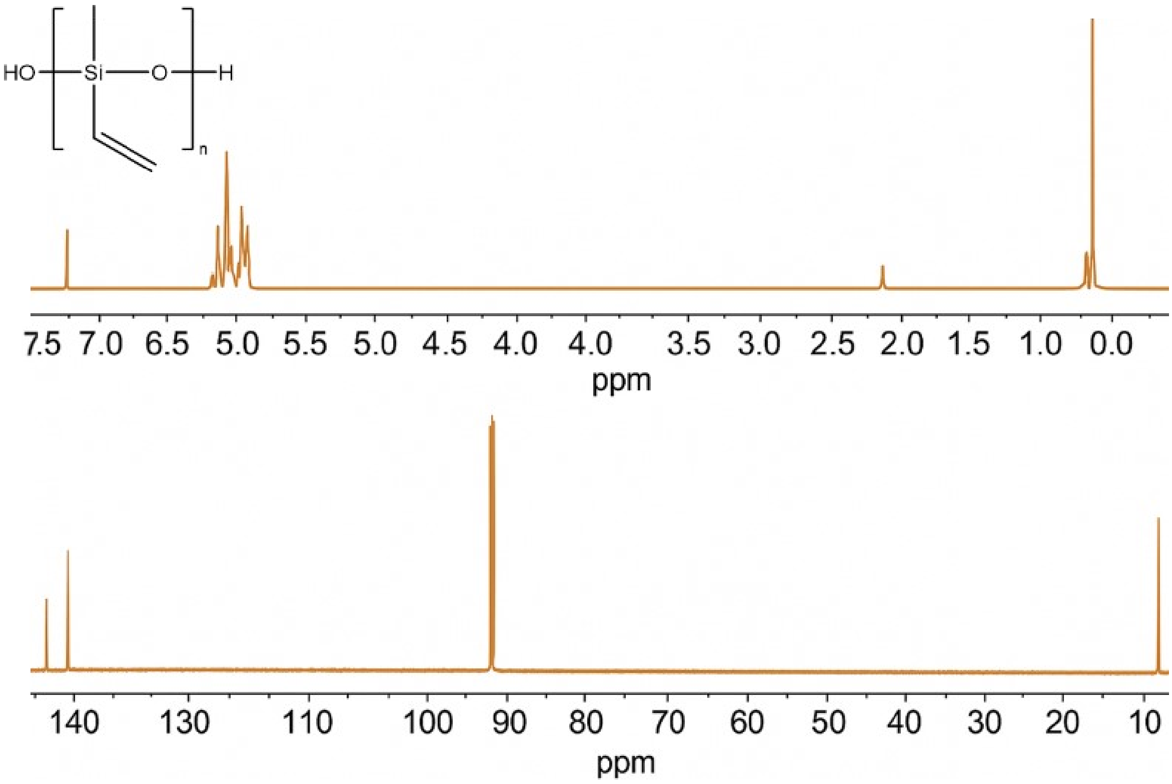

Fig. S1 1H and 13C NMR spectra of PV used for the synthesis of different materials.

GPC

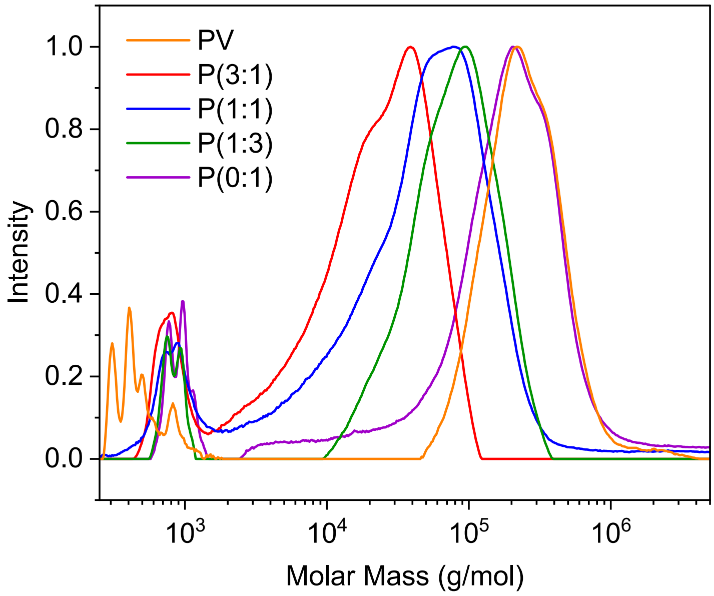

Fig. S2 GPC elugrams of the polymers PV, P(3:1), P(1:1), P(1:3), P(0:1). The polymer (PV) was synthesized through anionic ring-opening polymerization of 1,3,5,7-tetravinyl-1,3,5,7-tetramethylcyclotetrasiloxane using TMAH as the initiator. The PV contains a small amount of cycles which are difficult to remove by precipitation or distillation. GPC elugram of the P(1:0) could not be recorded as it is insoluble in THF.

Table S1 The molar mass and molar mass distributions results from GPC characterization.

Sample	M_n [g mol^{-1}]	M_w [g mol^{-1}]	PDI	Oligomer/cycles [wt.%]
PV	193900	296000	1.53	12.25
P(1:0)	NA	NA	NA	3.17
P(3:1)	4800	27400	5.71	9.69
P(1:1)	7900	65500	8.29	8.56
P(1:3)	51100	97200	1.90	7.53
P(0:1)	68800	245500	3.57	8.97

Synthesis

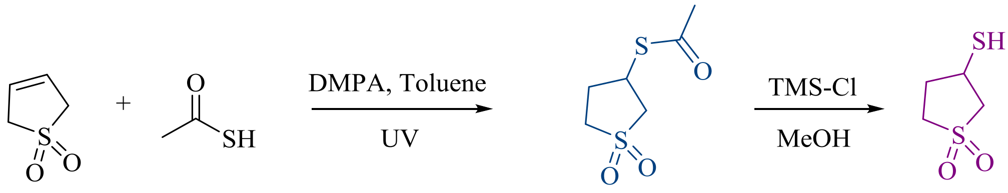

(1) (2)

Scheme 1: Synthesis of the 3-Thioacetylsulfolane (1) followed by reduction to 3-Mercaptosulfolane (2)

3-Thioacetylsulfolane (1)

To a solution of 3-sulfolane (1) (30.9 g, 261 mmol, 1 eq.) in toluene (330 ml), thioacetic acid (44.0 g, 578 mmol, 2.2 eq.) and DMPA (1.4 g, 5.5 mmol, 0.02 eq.) were added. The reaction mixture was irradiated with a UV lamp for 30 minutes, keeping the temperature below 50 °C. The mixture was then cooled to −10 °C using an ice/salt bath, and the resulting precipitate was filtered and washed with cold toluene to yield the compound as a white solid. (27.69 g, 54 %). ^1H NMR (400 MHz, $CDCl_3$, δ): 4.18–4.11 (m, 1H, CH–S), 3.54 (dd, J = 13.5 Hz, 8.0 Hz, 1H, CH–CH_2–SO_2), 3.28–3.21 (m, 1H, CH_2–CH_2–SO_2), 3.11 (ddd, J = 13.25 Hz, 7.6 Hz, 0.6 Hz, 1H, CH_2–CH_2–SO_2), 2.97 (dd, J = 13.5 Hz, 9.0 Hz, 1H, CH–CH_2SO_2), 2.63–2.55 (m, 1H, CH_2–CH_2–SO_2), 2.25–2.15 (m, 1H, CH_2–CH_2–SO_2); ^{13}C NMR (100 MHz, $CDCl_3$, δ): 194.0 (CO–S), 56.2 (CH–CH_2–SO_2), 51.6 (CH_2–CH_2–SO_2), 37.7 (CH–S), 30.6 (CH_3–CO), 29.2 (CH_2–CH_2–SO_2).

3-Mercaptosulfolane (2)

3-Thioacetylsulfolane (**1**) (27.50 g, 142 mmol, 1 eq.) was stirred in a mixture of THF (120 ml) and MeOH (90 ml) at 40 °C until all white solids disappeared. Then, TMS-Cl (0.62 g, 5.66 mmol, 0.04 eq.) was added, and the reaction mixture was stirred at 50 °C for 3 days. After removing the solvent under reduced pressure, the residue was dissolved in CH_2Cl_2 and washed with a saturated aqueous solution of $NaHCO_3$. The aqueous phase was thoroughly extracted with CH_2Cl_2, the combined organic phases were dried over $MgSO_4$, filtered, and the solvent was evaporated under reduced pressure. The residue was distilled at 1.2 mbar and 143 °C to yield compound **2** as a colorless liquid. (13.76 g, 64 %). ^1H NMR (400 MHz, $CDCl_3$, δ): 3.64–3.56 (m, 1H, CH–SH), 3.54 (dd, J = 13.3 Hz, 7.3 Hz, 1H, CH–CH_2–SO_2), 3.37–3.31 (m, 1H, CH_2–CH_2–SO_2), 3.10 (dddd, J = 13.3 Hz, 9.8 Hz, 7.7 Hz, 0.3 Hz, 1H, CH_2–CH_2–SO_2), 2.98 (dd, J = 13.2 Hz, 9.6 Hz, 1H, CH–CH_2–SO_2), 2.69–2.61 (m, 1H, CH_2–CH_2–CH), 2.16 (ddt, J = 13.57 Hz, 8.23 Hz, 9.90 Hz, 1H, CH_2–CH_2–CH), 2.02 (d, J = 7.4 Hz, 1H, SH); ^{13}C NMR (100 MHz, $CDCl_3$, δ): 60.1 (CH–CH_2SO_2), 52.4 (CH_2–CH_2–SO_2), 34.0 (CH_2–CH_2–CH), 33.8 (CH–SH).

NMR Spectra

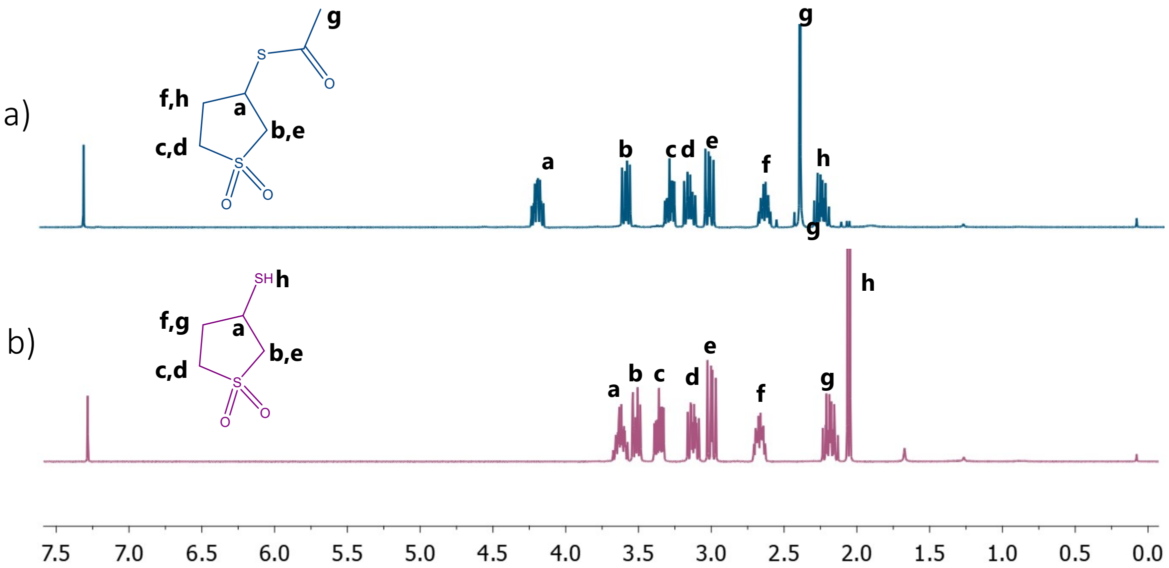

Fig. S3 ^{1}H NMR spectra of the 3-thioacetylsulfolane (a), and 3-mercaptosulfolane (b)

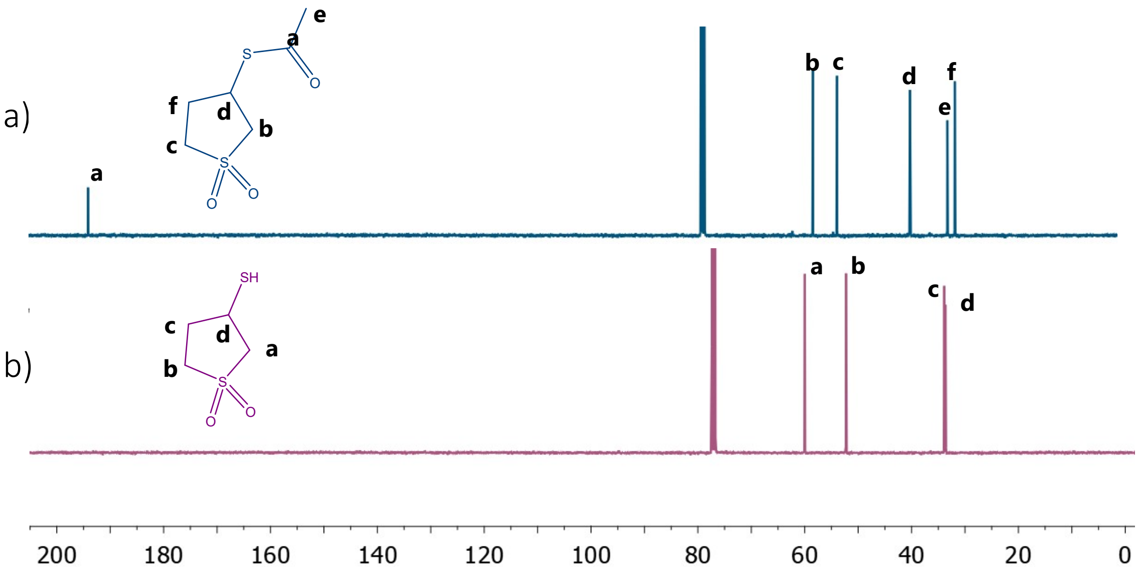

Fig. S4 ^{13}C NMR spectra of the 3-thioacetylsulfolane (a), and 3-mercaptosulfolane (b)

TGA

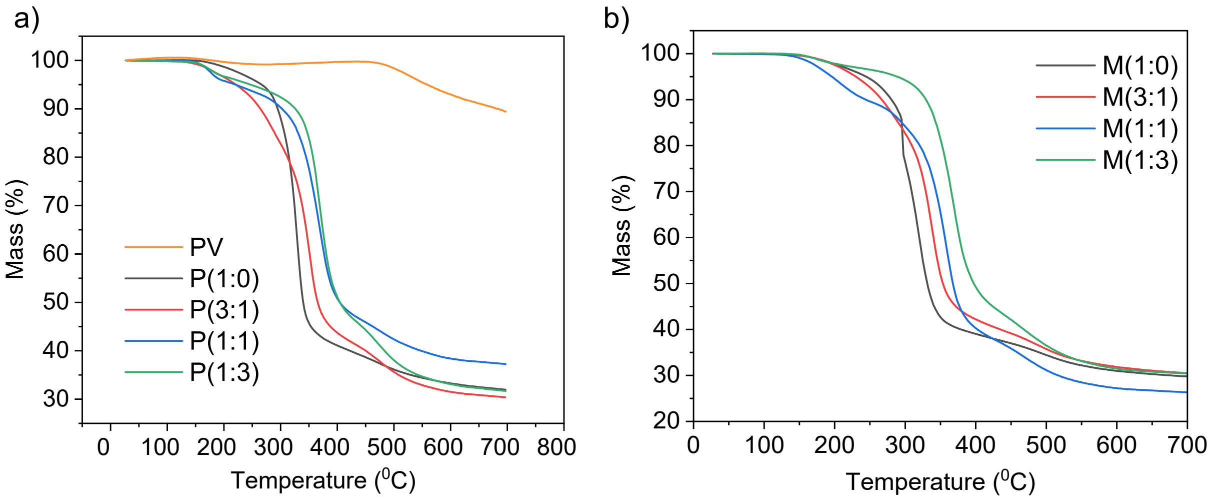

Fig. S5 TGA of the polymers **P(x:y)** (a), and materials **M(x:y)** (b)

Uniaxial Cyclic Test of M(3:1) and M(1:0)

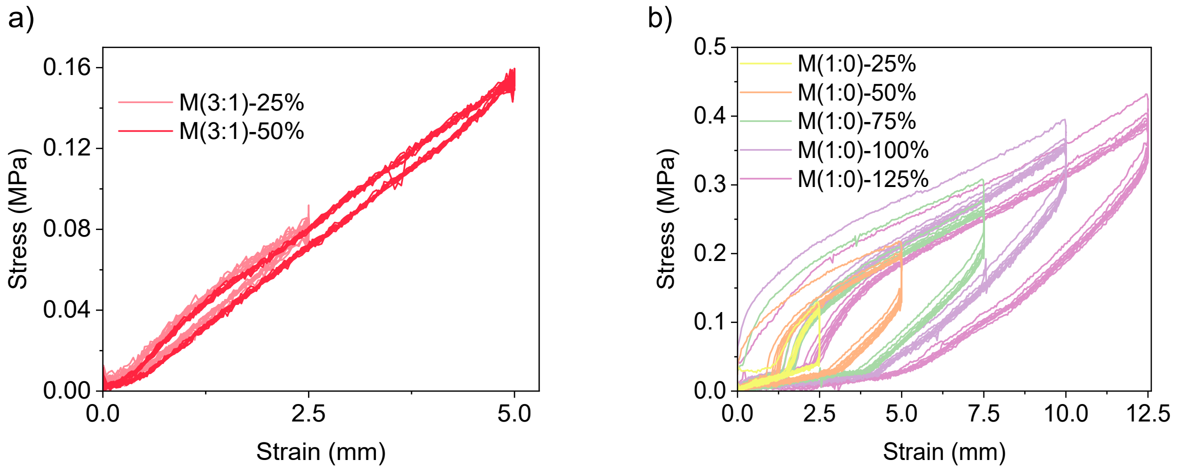

Fig. S6 Uniaxial cyclic test of **M(3:1)** at 25 %, 50 % strain for 10 cycles (a) and of **M(1:0)** at 25 %, 50 %, 75 %, 100 %, 125 % strain for 10 cycles (b).

Uniaxial cyclic tensile tests of the **M(1:0)** and **M(3:1)** samples were conducted at varying strain levels for ten cycles, with each measurement performed on new samples. **M(1:0)** displayed maximum strain of 125%. However, it exhibited inelastic deformation after the first cycle, though it subsequently followed the same trend in the following cycles for each test. The material **M(3:1)** showed elastic deformation at 25 % and 50 % strains.

Temperature Dependent Dielectric Permittivity Measurements

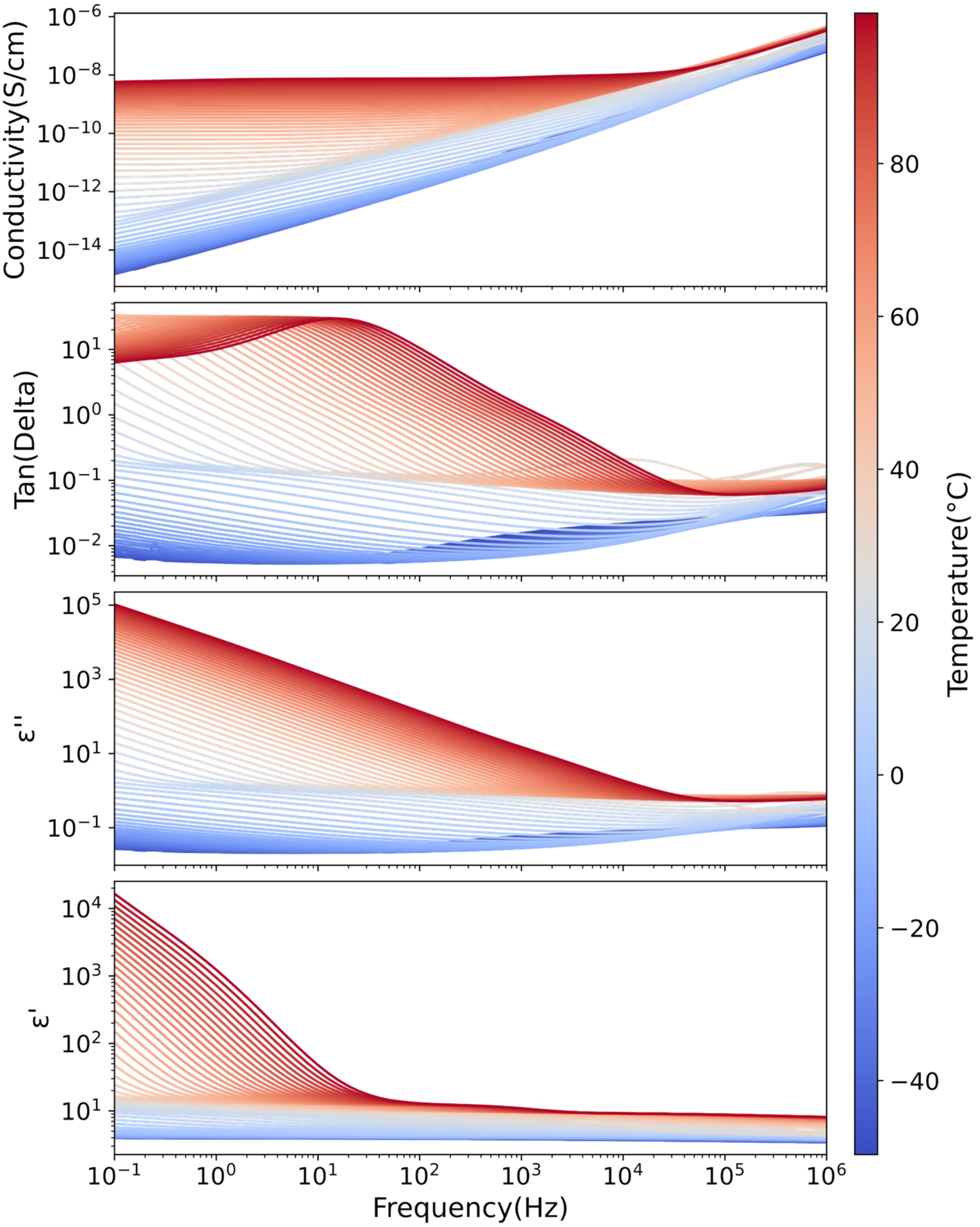

Fig. S7 The conductivity (σ'), loss tangent (tan (δ)), dielectric loss (ε''), and dielectric permittivity (ε') analysis at altering temperature as a function of frequency ranging from 10^{-1} and 10^{6} Hz for **M(1:0)**.

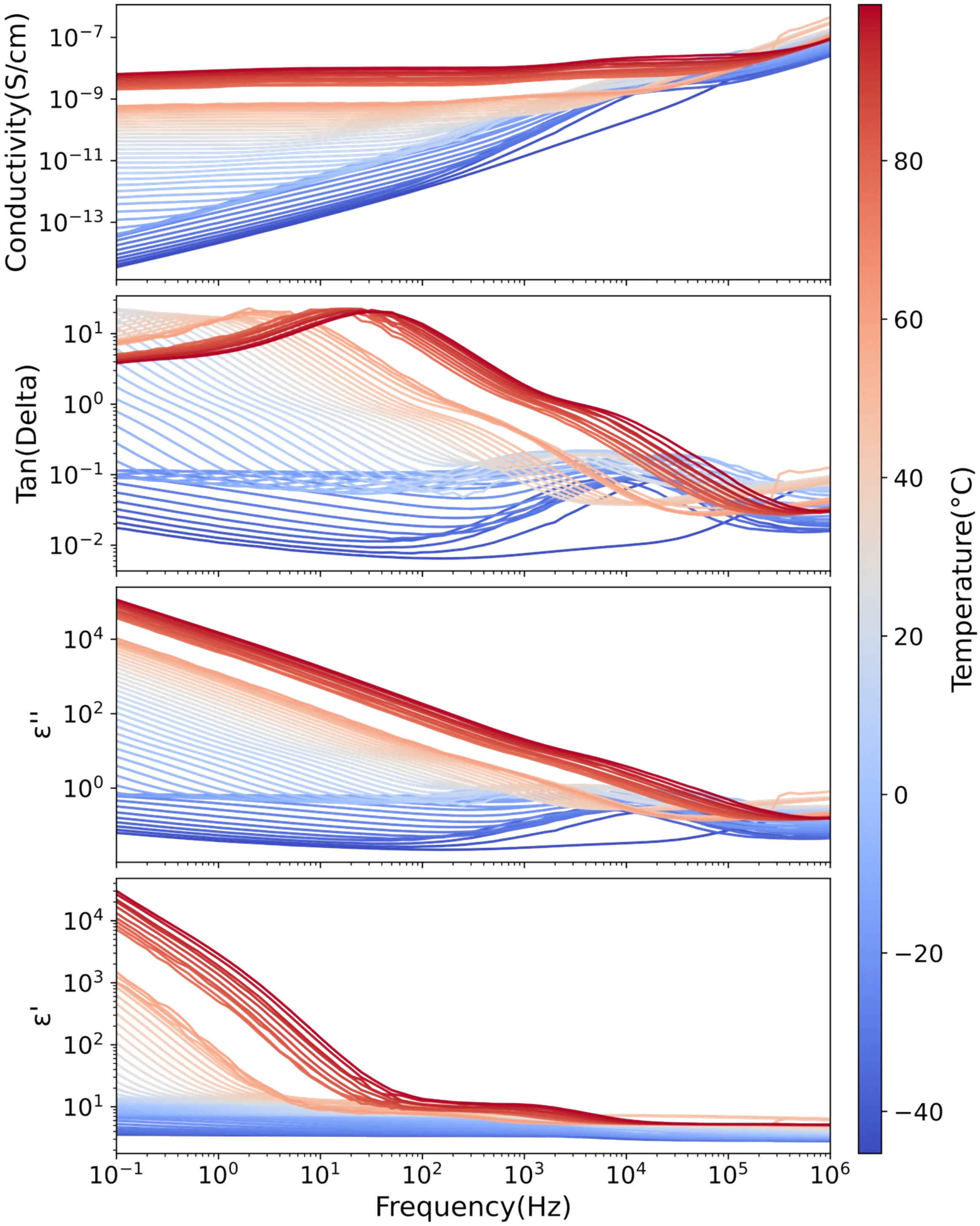

Fig. S8 The conductivity (σ'), loss tangent (tan (δ)), dielectric loss (ε''), and dielectric permittivity (ε') analysis at altering temperature as a function of frequency ranging from 10^{-1} and 10^{6} Hz for **M(3:1)**.

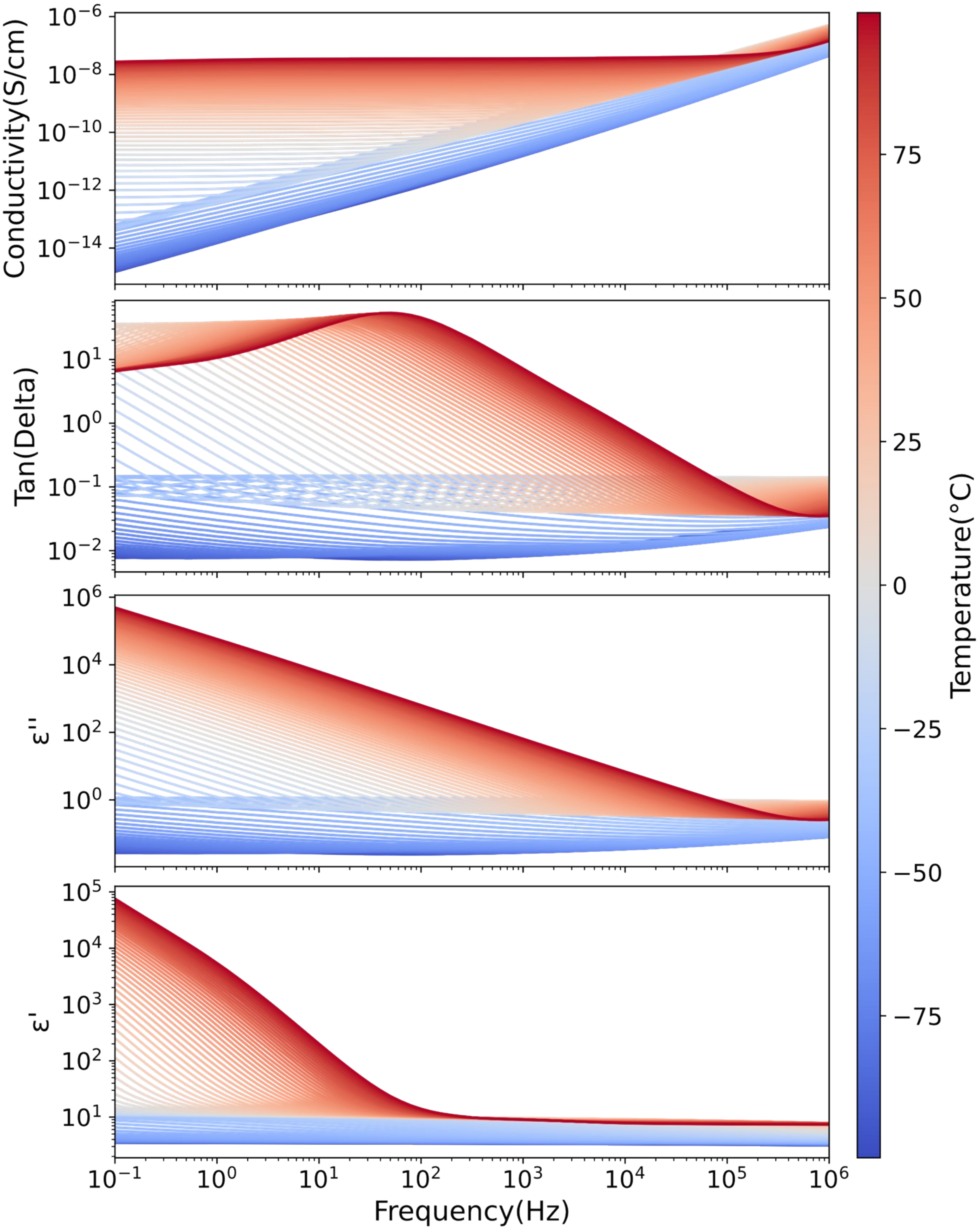

Fig.S9 The conductivity (σ'), loss tangent (tan (δ)), dielectric loss (ε''), and dielectric permittivity (ε') analysis at altering temperature as a function of frequency ranging from 10^{-1} and 10^{6} Hz for **M(1:1)**.

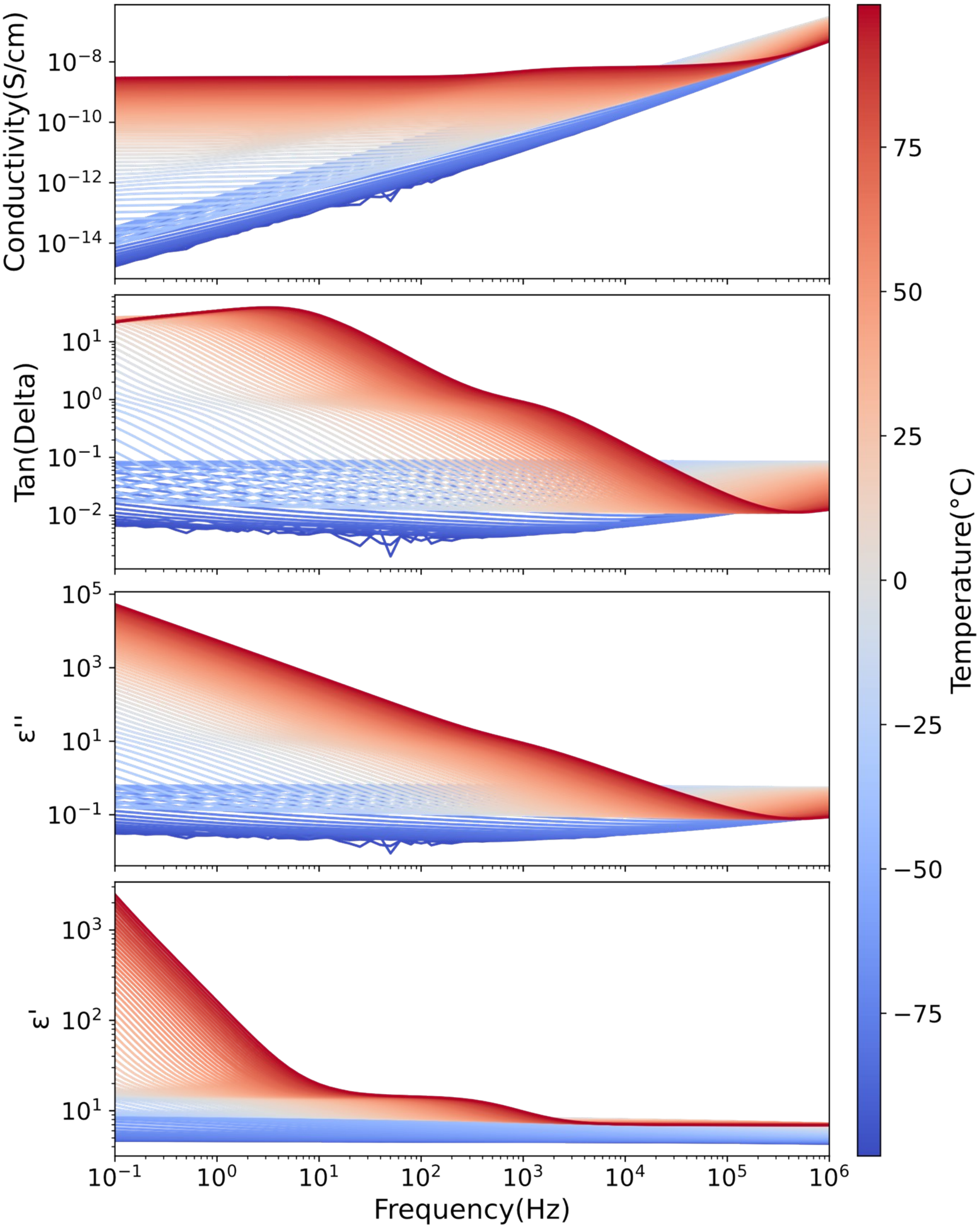

Fig. S10 The conductivity (σ'), loss tangent (tan (δ)), dielectric loss (ε''), and dielectric permittivity (ε') analysis at altering temperature as a function of frequency ranging from 10^{-1} and 10^{6} Hz for **M(1:3)**.

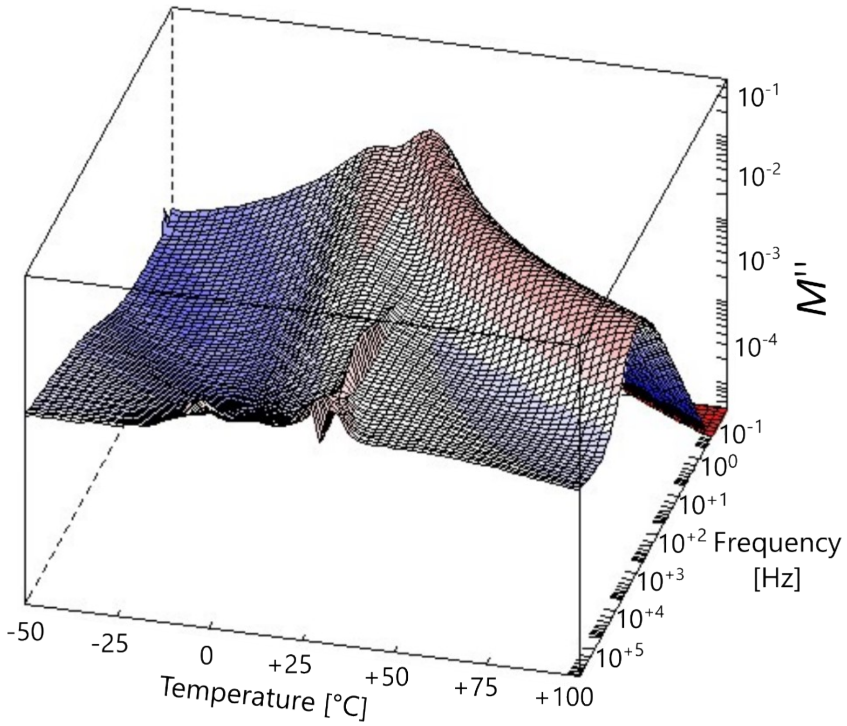

Fig. S11 3D plot of electric loss modulus M′′ of **M(1-0)**.

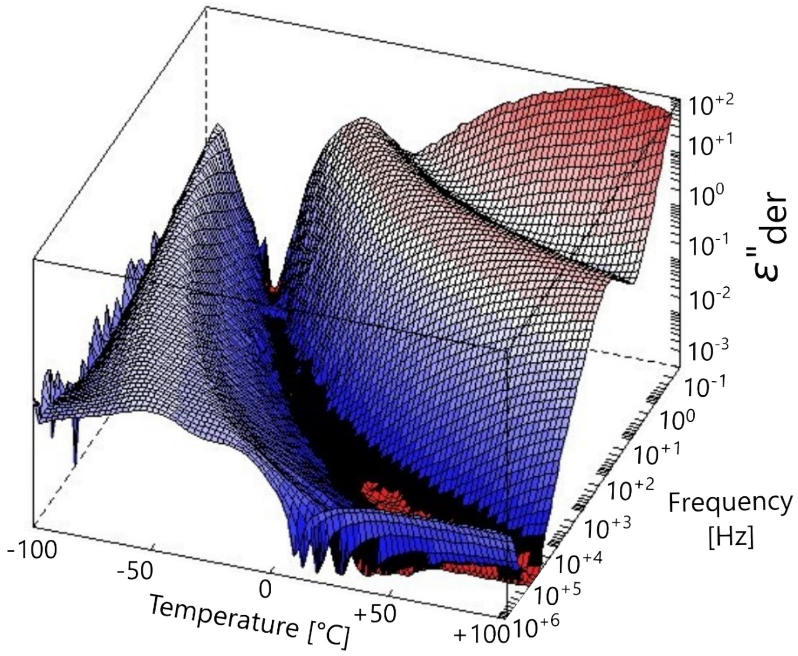

Fig. S12 3D plot of conduction-free ε″der curves of a **M(0:1)** as a function of frequency from 10^{-1} to 10^{6} Hz and temperatures from −100 to +100 °C.

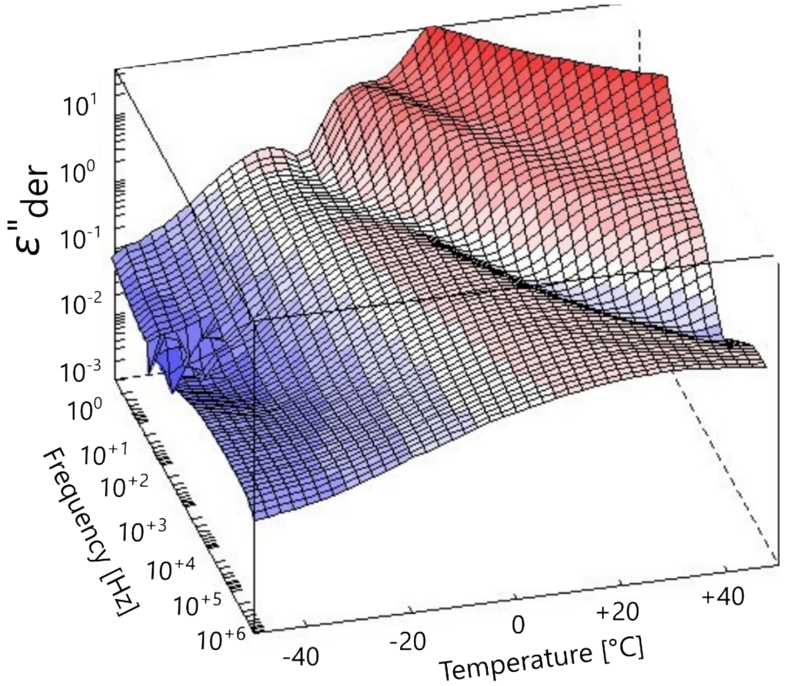

Fig. S13 3D plot of conduction-free ε″der curves of a 3-mercaptosulfolane functionalized V_4 (tetravinyltetramethylcyclotetrasiloxane) sample as a function of frequency from 10^{-1} to 10^6 Hz and temperatures from −100 to +100 °C.

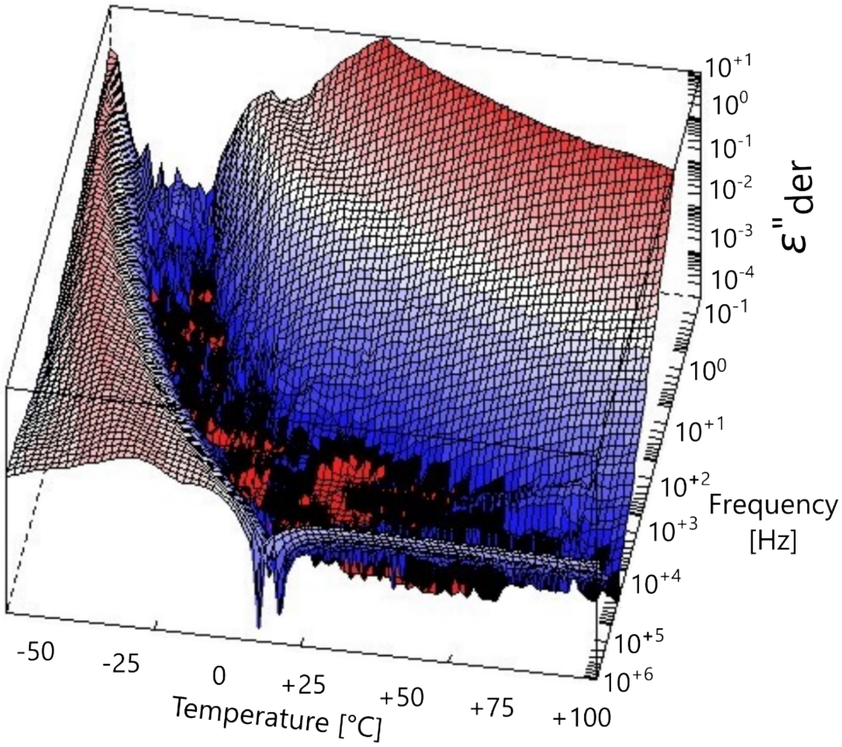

Fig. S14 3D plot of conduction-free ε″der curves of a butane thiol functionalized V_4 (tetravinyltetramethylcyclotetrasiloxane) sample as a function of frequency from 10^{-1} to 10^6 Hz and temperatures from −100 to +100 °C.

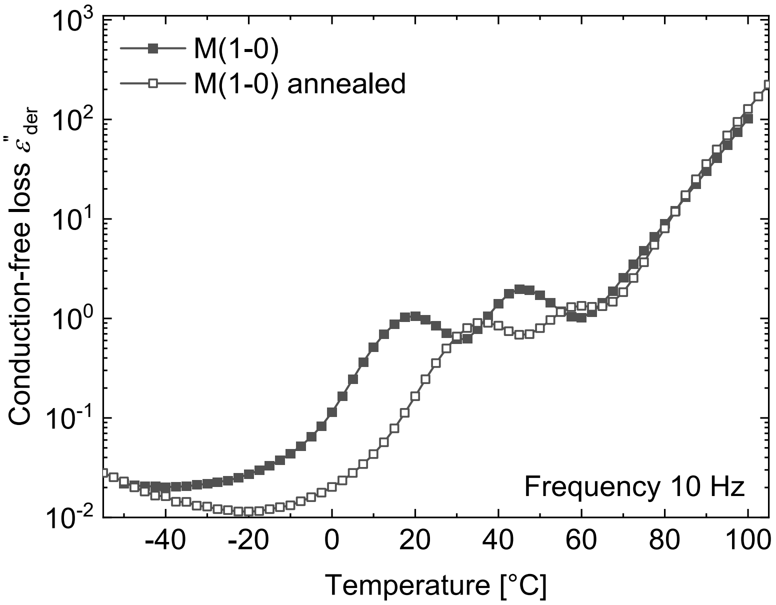

Fig. S15 ε''_{der} curves of a **M(1-0)** as a function of temperature at a frequency of 10 Hz before and after annealing.

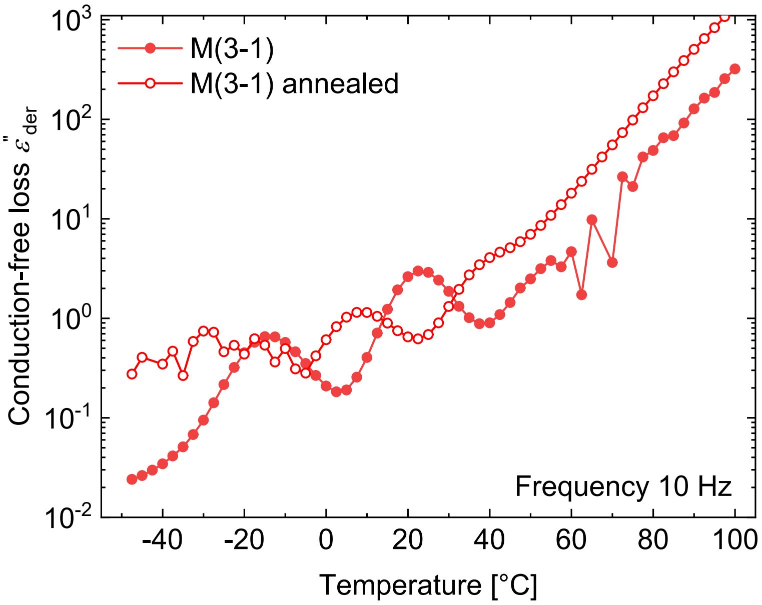

Fig. S16 ε''_{der} curves of a **M(3-1)** as a function of temperature at a frequency of 10 Hz before and after annealing.

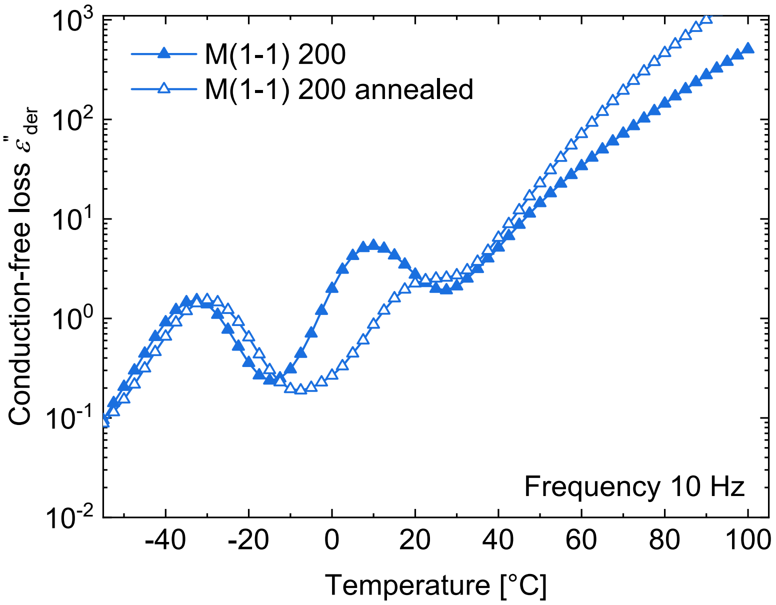

Fig. S17 ε''_{der} curves of a **M(1-1)-200** as a function of temperature at a frequency of 10 Hz before and after annealing

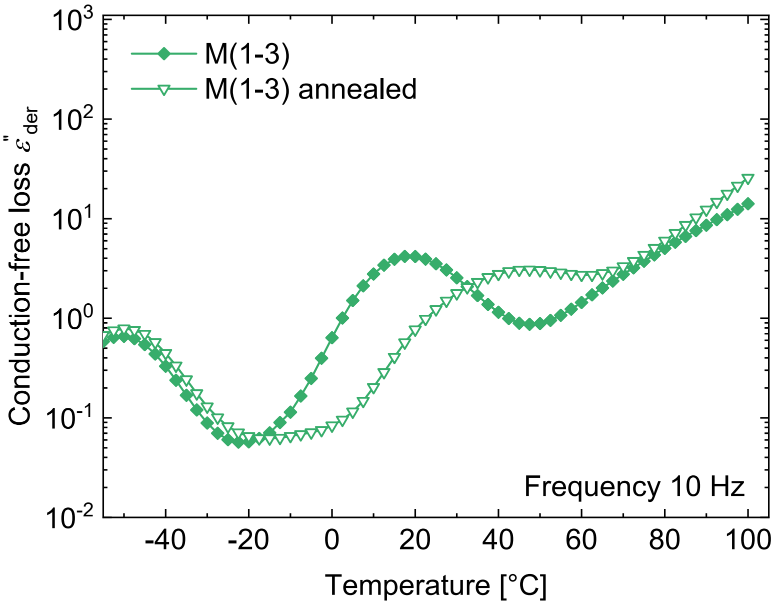

Fig. S18 ε''_{der} curves of a **M(1-3)** as a function of temperature at a frequency of 10 Hz before and after annealing.

Dielectric Breakdown

Table S2 Sample thickness, breakdown voltage, and calculated breakdown field for 10 individual samples of **M(1-1)**

**M(1-1)**		
Thickness (μm)	Breakdown Voltage (V)	Breakdown Field (V/ μm)
95	2300	24.21
95	1800	18.95
95	2750	28.94
95	2900	30.53
95	3400	35.79
95	2000	21.05
80	1500	18.75
80	3200	40
80	2400	30
80	1800	22.5

Table S3 Sample thickness, breakdown voltage, and calculated breakdown field for 10 individual samples of **M(1-1)-200**

**M(1-1)-200**		
Thickness (μm)	Breakdown Voltage (V)	Breakdown Field (V/ μm)
130	2300	17.69
130	1700	13.68
90	1700	18.89
90	3600	40
130	3900	30
130	5000	38.46
130	3600	27.69
130	3300	25.38
120	2700	22.5
130	3000	23.08

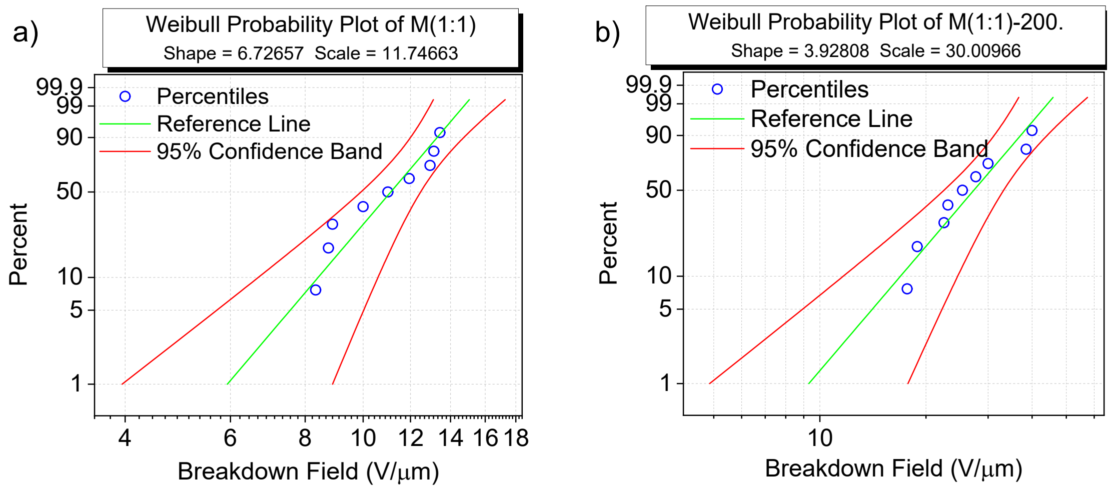

Fig. S19 Weibull probability plot of **M(1:1)** (a), and **M(1:1)-200** (b)

Tensile Test and DMA of M(1:1)-200

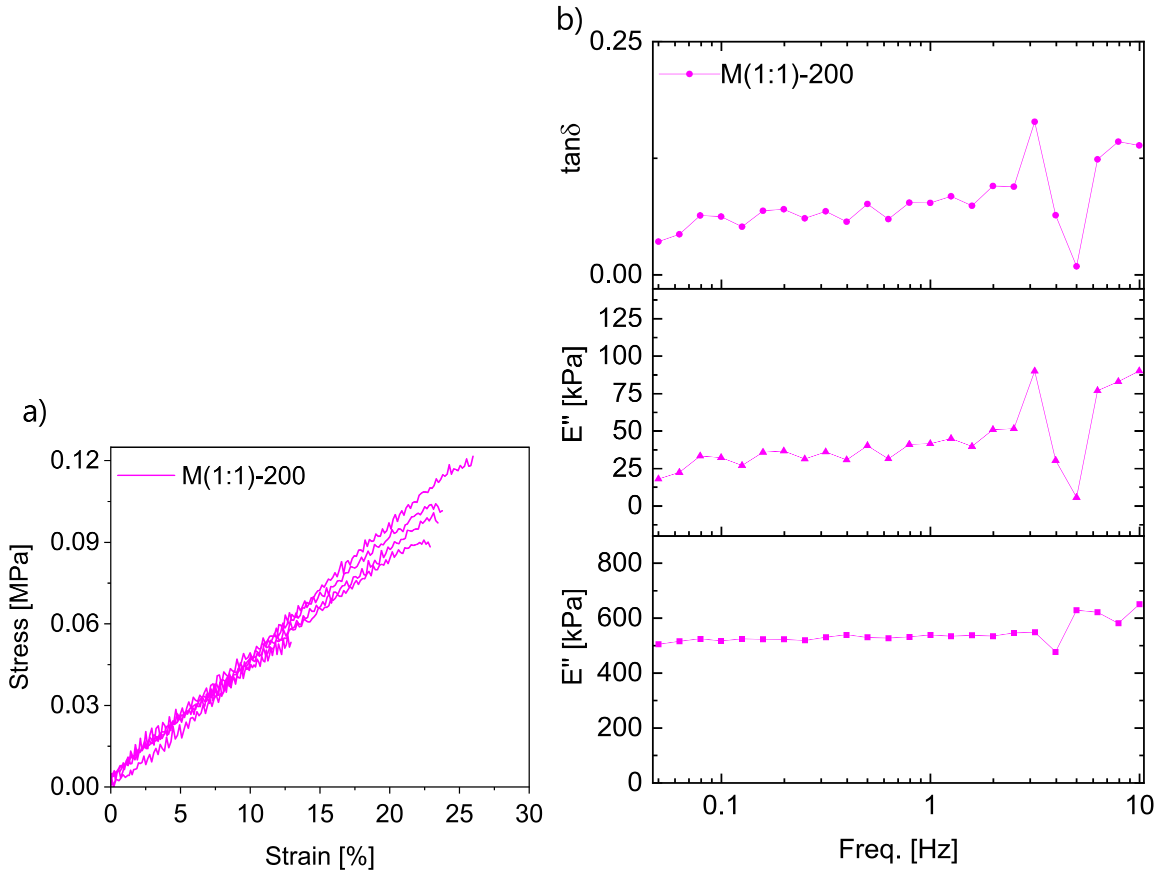

Fig.S20 Tensile test (a) and DMA (b) of **M(1:1)-200**.

Table S4 Key parameters for mechanical behavior of **M(1:1)-200**

	Cross-linker [mmol]	Thickness [μm]	$Y_{\%10}$ [kPa]	ϵ' @1 kHz	ϵ' @1 MHz
**M(1:1)-200**	1.204	85	430 $\pm$ 25	11.16	8.76

Impedance of M(1:1)-200

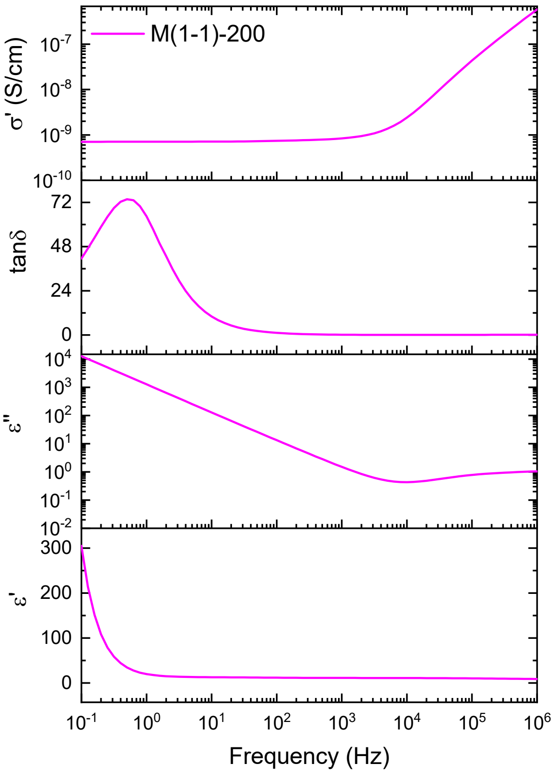

Fig. S21 Impedance spectroscopy at room temperature and different frequencies of **M(1:1)-200**.

Actuation test

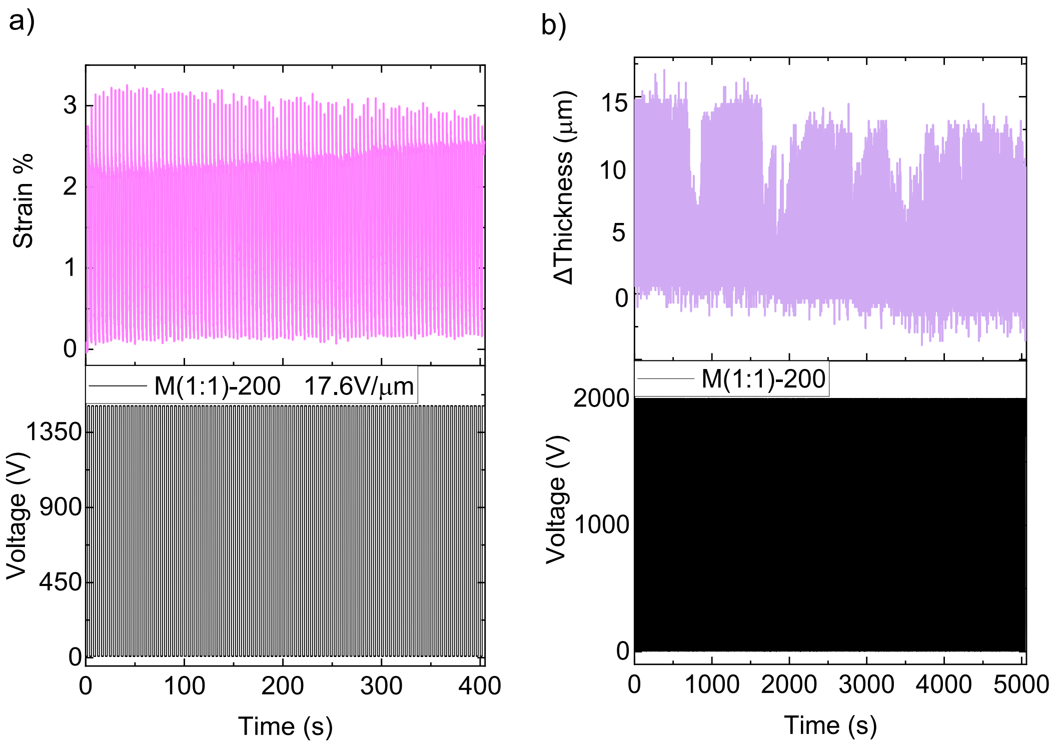

Fig. S22 Cyclic actuation test of a single layer **M(1:1)-200** for 100 cycles at 17.6 V μm^{-1} (1500 V) at 0.25 Hz (a), and stack actuator with five active layers with a thickness of ~145 μm at an electric field of 13.8 V μm^{-1} (2000 V) for more than 1000 cycles.

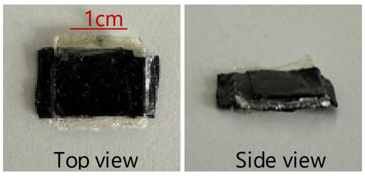

Fig. 23 Photo of the stack actuator constructed by layering an interdigitated structure composed of alternating 5 dielectric films (145 μm thick and a size of 1 × 1.5 cm) and 6 electrode layers (205 μm thick and a size of 1 × 1.5 cm).

A single layer of M(1:1)-200 with an 85 μm thickness was tested at 1500 V and 0.25 Hz for 100 cycles. The material exhibited a maximum actuation strain of 3.2%. The stack actuator constructed from the same material was tested for over 1000 cycles at 2000 V and 0.25 Hz. It can be observed that the actuation intermittently stopped but then resumed, continuing the actuation process.
